# A new wave of innovations within the DNA damage response

**DOI:** 10.1038/s41392-023-01548-8

**Published:** 2023-09-08

**Authors:** Qi Li, Wenyuan Qian, Yang Zhang, Lihong Hu, Shuhui Chen, Yuanfeng Xia

**Affiliations:** Domestic Discovery Service Unit, WuXi AppTec, 200131 Shanghai, China

**Keywords:** Drug development, Drug development

## Abstract

Genome instability has been identified as one of the enabling hallmarks in cancer. DNA damage response (DDR) network is responsible for maintenance of genome integrity in cells. As cancer cells frequently carry DDR gene deficiencies or suffer from replicative stress, targeting DDR processes could induce excessive DNA damages (or unrepaired DNA) that eventually lead to cell death. Poly (ADP-ribose) polymerase (PARP) inhibitors have brought impressive benefit to patients with *breast cancer gene (BRCA)* mutation or homologous recombination deficiency (HRD), which proves the concept of synthetic lethality in cancer treatment. Moreover, the other two scenarios of DDR inhibitor application, replication stress and combination with chemo- or radio- therapy, are under active clinical exploration. In this review, we revisited the progress of DDR targeting therapy beyond the launched first-generation PARP inhibitors. Next generation PARP1 selective inhibitors, which could maintain the efficacy while mitigating side effects, may diversify the application scenarios of PARP inhibitor in clinic. Albeit with unavoidable on-mechanism toxicities, several small molecules targeting DNA damage checkpoints (gatekeepers) have shown great promise in preliminary clinical results, which may warrant further evaluations. In addition, inhibitors for other DNA repair pathways (caretakers) are also under active preclinical or clinical development. With these progresses and efforts, we envision that a new wave of innovations within DDR has come of age.

## Introduction

Cells are constantly under DNA damage stress posed by endogenous or environmental agents.^1,2^ A complex DNA damage response (DDR) network has been evolved to maintain the integrity and fidelity of genomic DNA. These DDR networks include DNA repair pathways themselves and a repertoire of regulatory signaling events which are closely related to other cellular processes such as cell cycle, immunogenicity and apoptosis.^3–7^ Defects in DDR pathways or exposure to carcinogens can lead to accumulated DNA damage and genome instability, which could favor carcinogenesis.^8,9^ Disrupting DDR processes in cancer cells would aggregate genomic DNA damage and ultimately trigger senescence or programmed cell death.^1,7^ Now DNA repair defect has been validated as one of the targetable hallmarks in cancer.^10^

The scenarios for DDR inhibitors in clinic have been portrayed as: synthetic lethality, replication stress, and potentiation of chemo- or radio- therapy.^11^ Synthetic lethality is described as malfunction in one certain DDR mechanism renders cells more reliant on other somewhat redundant DDR pathways to survive.^12,13^ Hitherto synthetic lethality remains the only approved strategy in clinic for DDR targeting therapy, such as Poly (ADP-ribose) polymerase (PARP) inhibitors’ success in *breast cancer gene (BRCA)* mutation or homologous recombination deficient (HRD) solid tumors.^14^ Replication stress represents a phenomenon that DNA synthesis slows down or replication fork stalls in S phase, which is characterized by extended single strand DNA (ssDNA) exposure.^15–17^ In cancer cells, uncontrolled proliferation, deregulated cell cycle progression or exhausted dNTPs due to nucleotide analog chemotherapy treatment, would cause replication stress. To avoid more catastrophic genome instabilities due to replication stress, ssDNA-bound replication protein A (RPA) would activate the ataxia–telangiectasia and Rad3 related (ATR) - checkpoint kinase 1 (CHK1) - Wee1-like protein kinase (WEE1) - cyclin dependent kinase 1 or 2 (CDK1/2) axis to control the replication firing and arrest cell cycle progress.^18^ Albeit the intriguing potentiality to use replication stress as predictive biomarkers for ATR, CHK1 or WEE1 inhibitors, more indicative and predictive biomarkers are required to be verified for patient stratification in clinic.^11,19^ Combination with DNA damage inducing agents such as chemotherapy and radiation is the initial purpose of targeting DDR processes.^20,21^ However this strategy have been confounded for years because of overlapped toxicity, difficult to dosing, and intolerable damage to normal tissues.^22,23^

**Fig. 1:**
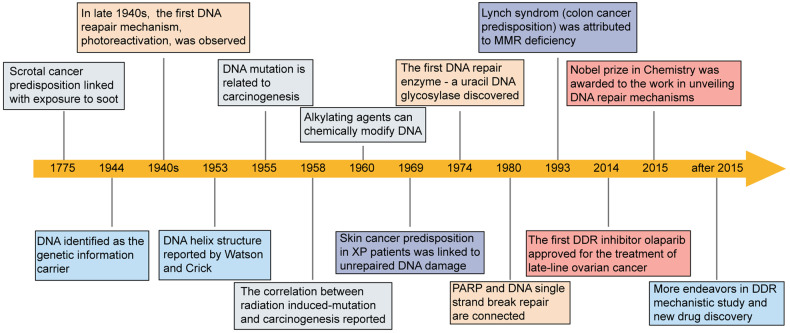
Timeline to show the selected key milestones in DDR mechanism identification, DDR correlation with carcinogenesis and drug discovery. As early as 1775, the linkage between cancer predisposition and environmental insult was observed. However, until 1940s to 1960s, the correlations of carcinogenesis and DNA damage induced by chemicals or radiation became clear with the emergence of molecular biology. Since 1970s, DDR pathways have been depicted as a spectrum of catalytic processes, protein-protein interactions and protein-DNA interactions. Deficiencies in DDR pathways could facilitate carcinogenesis, and can be targeted by small molecule drugs, such as PARP inhibitors’ approval for the treatment of *BRCA* mutant ovarian cancer. All these efforts eventually led to the grant of Nobel Prize in Chemistry in DDR area in 2015. Now a great deal of interest has been evoked for the study of DDR mechanisms as well as antitumor drug discovery

The first-ever DNA repair inhibitor, PARP inhibitor olaparib, was approved in 2014 for the late line treatment of *BRCA* deficient ovarian cancer^24^ (Fig. 1). Hitherto at least 6 PARP inhibitors have been launched worldwide, and the indications have been expanded to breast cancer, prostate cancer and pancreatic cancer^25^ (Table 1). Now the PARP inhibitor development strategy has moved to selectively inhibiting PARP1 which could maintain the efficacy while mitigating side effects.^26,27^ Beyond PARP, a subset of DNA damage checkpoints have emerged as antitumor targets in clinic, including WEE1, ATR, CHK1, ataxia–telangiectasia mutated (ATM), checkpoint kinase 2 (CHK2), protein kinase membrane associated tyrosine/threonine 1 (PKMYT1), polo-like kinase 1 (PLK1) and even tumor suppressor p53 (Table 2). Inhibitors of WEE1, ATR, CHK1 and PLK1 have also achieved preliminary response in certain types of cancer patients. Recently, small molecule inhibitors of Polymerase theta (Polθ), DNA repair protein RAD51 homolog 1 (RAD51), ubiquitin carboxyl-terminal hydrolase 1 (USP1), poly (ADP-Ribose) glycohydrolase (PARG) and werner syndrome helicase (WRN) were reported, some of which have moved into clinical investigations (Table 2). Concerning the DDR mechanisms and inhibitors have been widely reviewed elsewhere,^10,28–32^ we embark on the newly progress and recently identified DDR targets and inhibitors in this manuscript. Due to the span of our knowledge, we cannot cover all the progress of targets and inhibitors within DDR space. However inspired by these intriguing progresses and findings, we prospect a new wave of innovations within DDR targets in the near future.

**Table 1:** Approved indications of PARP inhibitors (based on the most updated labels)

Drug	Cancer types	Indication	Biomarkers
Olaparib	Ovarian cancer	First-line maintenance; monotherapy	Complete or partial response to first-line platinum-based chemotherapy; *BRCA* mutant
		First-line maintenance; combined with bevacizumab	Complete or partial response to first-line platinum-based chemotherapy; HRD positive
		Second-line maintenance; monotherapy	Complete or partial response to platinum-based chemotherapy
	Brest cancer	Adjuvant therapy for HER2-negative breast cancer patients who have received neoadjuvant or adjuvant chemotherapy; monotherapy	*BRCA* mutant
		Chemotherapy pre-treated (neoadjuvant, adjuvant or metastatic setting) HER2-negative metastatic breast cancer; monotherapy	*BRCA* mutant
	Pancreatic cancer	First-line maintenance; monotherapy	Disease has not progressed on at least 16 weeks of a first-line platinum-based chemotherapy; *BRCA* mutant
	Prostate cancer	Pre-treated metastatic castration-resistant prostate cancer; monotherapy	HR repair gene muatant
Rucaparib	Ovarian cancer	Second-line maintenance; monotherapy	Complete or partial response to platinum-based chemotherapy; *BRCA* mutant
	Prostate cancer	Androgen receptor-directed therapy and a taxane-based chemotherapy pretreated metastatic castration-resistant prostate cancer; monotherapy	*BRCA* mutant
Niraparib	Ovarian cancer	First-line maintenance; monotherapy	Complete or partial response to platinum-based chemotherapy;
		Second-line maintenance; monotherapy	Complete or partial response to platinum-based chemotherapy; *BRCA* mutant
Talazoparib	Breast cancer	Pre-treated HER2-negative locally advanced or metastatic cancer metastatic breast cancer; monotherapy	*BRCA* mutant
Pamiparib	Ovarian cancer	Pre-treated patients; monotherapy	*BRCA* mutant
Fluzoparib	Ovarian cancer	Pre-treated patients; monotherapy	Platinum-sensitive; *BRCA* mutant
		Second-line maintenance; monotherapy	Platinum-sensitive; *BRCA* mutant

**Table 2:** Notable clinical-stage DDR inhibitors and clinical trials in active development^a^

Pathway	Target	Drug	Combination	Phase	Cancer type	Clinical Trial Identifier
SSBR	PARP1-selective	NMS-03305293	Monotherapy	1	ASTs^b^	NCT04182516
			Temozolomide	1/2	Glioma, glioblastoma	NCT04910022
		AZD5305	Monotherapy; Paclitaxel; Carboplatin; Trastuzumab Deruxtecan; Datopotamab Deruxtecan	1/2	ASTs	NCT04644068
			Ceralasertib	1/2	ASTs	NCT02264678
			Datopotamab deruxtecan or Durvalumab + Datopotamab deruxtecan	2	ASTs	NCT05489211
			Enzalutamide; Abiraterone acetate; Darolutamide	1/2	Metastatic prostate cancer	NCT05367440
		AZD9574	Monotherapy; Temozolomide	1/2	ASTs, breast cancer, glioma	NCT05417594
DSBR	Polθ	ART4215	Monotherapy; Talazoparib; Niraparib	1/2	ASTs	NCT04991480
	RAD51	CYT0851	Monotherapy; Gemcitabine; Capecitabine; Rituximab and Bendamustine	1/2	ASTs, B-Cell Malignancies	NCT03997968
TLS and FA	USP1	KSQ4279	Monotherapy; an oral PARP inhibitor; a platinum-based chemotherapy	1	ASTs	NCT05240898
DNA damage checkpoint	ATR	Berzosertib	Topotecan	2	Small cell lung cancer	NCT04768296
				2	Small cell cancers	NCT03896503
			Irinotecan	2	Gastric or Gastroesophageal Junction Cancer	NCT03641313
				1	ASTs	NCT02595931
			Gemcitabine + Carboplatin	2	Urothelial carcinoma	NCT02567409
				1	Ovarian cancer	NCT02627443
			Gemcitabine	2	Leiomyosarcoma	NCT04807816
			Carboplatin	2	Castration-resistant prostate carcinoma	NCT03517969
			Lurbinectedin	1/2	Small cell lung cancer; ASTs	NCT04802174
			Radiation	1	HER2-negative breast carcinoma	NCT04052555
				1	Brain metastases	NCT02589522
			Radiation + Cisplatin	1	Head and Neck Cancer	NCT02567422
			Pembrolizumab + Gemcitabine + Carboplatin	1/2	Nonsmall cell lung squamous carcinoma	NCT04216316
			Avelumab	1/2	ASTs	NCT04266912
			Sacituzumab Govitecan	1/2	Small cell lung cancer; ASTs	NCT04826341
		Ceralasertib	Monotherapy	2	*ATM* mutant ASTs	NCT04564027
			Durvalumab	3	Nonsmall cell lung cancer	NCT05450692
				1	ASTs	NCT05514132
				2	Melanoma	NCT05061134
			Durvalumab + chemotherapy	2	Small cell lung cancer	NCT04699838
				2	Triple negative breast cancer	NCT05582538
			Olaparib	2	Osteosarcoma	NCT04417062
				2	*IDH1* and *IDH2* mutant tumors	NCT03878095
				2	*BRCA* mutant breast cancer	NCT04090567
				2	Prostate cancer	NCT03787680
			Durvalumab; Olaparib; monotherapy	2	ASTs	NCT03682289
			Olaparib; Durvalumab; AZD5305; Carboplatin	1/2	ASTs	NCT02264678
			Trastuzumab deruxtecan	1	ASTs	NCT04704661
		Elimusertib	Monotherapy	1/2	ASTs	NCT05071209
				1	ASTs and lymphoma	NCT03188965
			Niraparib	1	ASTs	NCT04267939
			Pembrolizumab	1	ASTs	NCT04095273
			Pembrolizumab + Radiation	1	ASTs	NCT04576091
			Irinotecan + 5-fluorouracil + Leucovorin	1	ASTs	NCT04535401
			Cisplatin; Cisplatin + Gemcitabine	1	ASTs	NCT04491942
			Irinotecan; Topotecan	1	ASTs	NCT04514497
			Gemcitabine	1	Ovarian cancer	NCT04616534
		Gartisertib	Niraparib	1	Ovarian cancer	NCT04149145
		Camonsertib	Monotherapy; Niraparib	1/2	ASTs	NCT04972110
			Monotherapy; Talazoparib or Gemcitabine	1/2	ASTs	NCT04497116
			Olaparib	1/2	Chronic lymphocytic leukemia	NCT05405309
			RP6306	1	ASTs	NCT04855656
			Radiation	1/2	ASTs	NCT05566574
		SC0245	Monotherapy	1	ASTs	CTR20210769
			Irinotecan	1/2	Small cell lung cancer	NCT05731518
		ART0380	Monotherapy; Gemcitabine; Irinotecan	1/2	ASTs	NCT04657068
		ATRN-119	Monotherapy	1/2	ASTs	NCT04905914
		IMP9064	Monotherapy	1	ASTs	NCT05269316
		LF0397	Monotherapy	1	ASTs	CTR20221402
	WEE1	Azenosertib	Monotherapy	1	Triple-negative breast cancer, Ovarian cancer	NCT05368506
				2	Uterine serous carcinoma	NCT04814108
				2	High-grade serous ovarian cancer	NCT05128825
				1	ASTs	NCT04158336
			Gemcitabine	1/2	Osteosarcoma	NCT04833582
			Niraparib	1/2	Ovarian cancer	NCT05198804
			Monotherapy; Encorafenib + Cetuximab	1/2	Colorectal cancer	NCT05743036
			Carboplatin; Pegylated liposomal doxorubicin; Paclitaxel; Gemcitabine	1	Ovarian cancer	NCT04516447
			Zn-C5	1/2	Acute myeloid leukemia	NCT05682170
		Debio0123	Monotherapy	1	ASTs	NCT05109975
			Carboplatin	1	ASTs	NCT03968653
			Temozolomide; Temozolomide + Radiotherapy	1/2	Glioblastoma	NCT05765812
		SC0191	Monotherapy	1	ASTs	CTR20210649
		IMP7068	Monotherapy	1	ASTs	NCT04768868
	ATM	AZD1390	Monotherapy	1	Glioblastoma	NCT05182905
			Radiation	1	Glioblastoma	NCT03423628
				1	ASTs	NCT05678010
				1	Soft tissue sarcoma	NCT05116254
				1	Nonsmall cell lung cancer	NCT04550104
		M4076	Monotherapy	1	ASTs	NCT04882917
	CHK1/2	Prexasertib	Monotherapy; Gemcitabine	1/2	Ovarian cancer, endometrial adenocarcinoma, urothelial carcinoma	NCT05548296
		LY2880070	Gemcitabine	1/2	ASTs	NCT02632448
				2	Ewing Sarcoma	NCT05275426
	PLK1	Onvansertib	Monotherapy	2	Small cell lung cancer	NCT05450965
				1	Chronic myelomonocytic leukemia	NCT05549661
			Irinotecan + Leucovorin + 5-fluorouracil + Bevacizumab	1/2	*KRAS* mutant colorectal cancer	NCT03829410
				2	*KRAS* mutant colorectal cancer	NCT05593328
			Nanoliposomal irinotecan + Leucovorin + 5-fluorouracil	2	Pancreatic ductal adenocarcinoma	NCT04752696
			Paclitaxel	1/2	HER2-negative breast cancer	NCT05383196
			Abiraterone + Prednisone	2	Castration-resistant prostate cancer	NCT03414034
		Plogosertib	Monotherapy	1	Leukemias, Myelodysplastic syndromes	NCT03884829
				1/2	ASTs, lymphoma	NCT05358379
	Aurora-A	Alisertib	Osimertinib	1	*EGFR* mutant lung cancer	NCT04085315
			Pembrolizumab	1/2	Rb-deficient head and neck squamous cell cancer	NCT04555837
		WJ05129	Monotherapy	1/2	ASTs	NCT05326035
		JAB-2485	Monotherapy	1/2	ASTs	NCT05490472
	PKMYT1	RP6306	Monotherapy; Camonsertib	1	ASTs	NCT04855656
			Irinotecan + Leucovorin + 5-fluorouracil	1	ASTs	NCT05147350
			Gemcitabine	1	ASTs	NCT05147272
				2	CDK4/6-inhibitor resistant ER+/HER2- metastatic breast cancer	NCT05601440
			Gemcitabine; Irinotecan + Leucovorin + 5-fluorouracil; Trastuzumab	2	ASTs	NCT05605509
	p53 Y220C	PC14586	Monotherapy	1/2	ASTs	NCT04585750

^a^First-generation PARP inhibitors & completed/withdrawn clinical trials not included

^b^ASTs: advanced solid tumors

## A historical perspective about DDR and cancer

In 1944, DNA was first-time identified as genetic information carrier (Fig. 1).^33^ About 10 years later in 1953, Watson and Francis Crick resolved the double helix structure of DNA,^34^ which laid a foundation for molecular biology as well as DDR mechanistic studies. From 1940s to 1960s, one type of direct reversal repair mechanism, photoreactivation to resolve cyclobutane pyrimidine dimers induced by ultraviolet (UV) exposure, was discovered.^35–37^ Then in 1970s, Tomas Lindahl observed the spontaneous decay of DNA which evoked the ground breaking identification of the first DNA repair enzyme, a uracil DNA glycosylase.^38–40^ Over the following decades, hundreds of proteins involved in kinds of DDR pathways, such as PARP (1980),^41^ DNA-dependent protein kinase catalytic subunit (DNA-PKcs, 1985),^42^ ATM (1995),^43^ CHK1 (1996),^44^ etc, were identified. In 2015, the Nobel Prize in Chemistry was granted to Tomas Lindahl, Paul Modrich and Aziz Sancar for their seminal study in DNA repair mechanism. Now the underlying mechanisms of DDR including protein-protein interaction, protein-nucleic acids interactions, catalytic processes, are still rapidly evolving.

The first evidence of the correlation between environmental insult and cancer can be traced back to 1775, when Percival Pott linked the predisposition of scrotal cancer to exposure to soot^45,46^ (Fig. 1). It was widely accepted by 1955 that chemical mutagens could lead to cancer susceptibility by increasing gene mutation rates.^47^ With the understanding of DNA chemical structure, Phil Lawley and Peter Brooks demonstrated that mustard gas and alkylating agents could form covalent DNA adducts, which impaired normal template functions.^48,49^ Shortly after that, they further illustrated that polynuclear aromatic hydrocarbons (also a component of tobacco smoke) exposure could result in DNA adducts and facilitate cancer initiation.^50^ This finding provides strong evidence for the linkage between chemical alterations in DNA and carcinogenesis. The correlation between radiation and cancer was only observed decades after the discovery of X-ray in 1895. A report in 1958 from United Nations Scientific Committee on the Effects of Atomic Radiation (UNSCEAR) concluded that in atomic bomb survivors, radiation-induced mutations were responsible for carcinogenesis^51^ (Fig. 1).

Defects in DDR genes accounted for dozens of hereditary diseases as well as carcinogenesis (Table 3). In 1969, Jim Cleaver linked the skin cancer predisposition of xeroderma pigmentosum (XP) to unrepaired DNA damage.^52^ XP patients developed skin cancer at the median age of 8 years^53^ (Fig. 1 and Table 3). Subsequently, these unrepaired DNA damages were attributed to mutation in NER genes. In 1990s, a colorectal cancer risk factor, Lynch syndrome, was shown to be related to familial mutations in mismatch repair (MMR) proteins^54–57^ (Fig. 1 and Table 3). Colorectal cancer patients with defective MMR (dMMR) are characterized by instabilities of simple repeated sequences in their genomes. dMMR has been widely known as microsatellite instability and used as a predictive biomarker for immunotherapy.^58,59^ Also in 1990s, women with familial mutations in genes *BRCA1* and *BRCA2* were found to be prone to breast cancer or ovarian cancer.^60–62^ Interestingly, familial mutations in *BRCA1* exhibit a different cancer spectrum from *BRCA2* mutations.^63^ *BRCA1* mutations are predominately implicated in breast and ovarian cancers,^64^ whereas *BRCA2* mutations are predisposed to breast, prostate, pancreatic, melanoma and ovarian cancers (Table 3). Nowadays *BRCA* mutations have been validated as biomarkers for patient selection for PARP inhibitors in clinic.^25^ Hitherto dozens of DDR genes have been identified to be associated with cancer predisposition (Table 3). Their potential use as biomarkers and/or antitumor targets are still under active exploitation.

**Table 3:** Examples of DDR gene mutation associated hereditary disease

Hereditary disease	Symptom	Related mutant genes	Cancer predispositions
Xeroderma Pigmentosum	Severe photosensitivity of the UV radiation-exposed regions of the skin; neurological abnormalities	*POLK, ERCC5, ERCC2, ERCC4, ERCC3, POLH, DDB2, XPA, XPB, XPC, XPD*	Skin cancers, angiomas, and sarcoma
Ataxia Telangiectasia	Early onset progressive cerebellar ataxia; oculocutaneous telangiectasia; weakened immune system and hypersensitivity to ionizing radiation	*ATM*	Leukemia and lymphoma
Nijmegen Breakage Syndrome	Short stature, distinctive facial features, recurrent respiratory tract infections, intellectual disability	*NBS1*	Lymphoma
Ataxia Telangiectasia-Like Disorder	Progressive cerebellar degeneration resulting in ataxia and oculomotor apraxia	*MRE11A*	Lymphoma
Seckel Syndrome	Growth retardation, very small head, blood abnormalities	*ATR*	Lymphomas, AML
Bloom Syndrome	Proportional dwarfing; Immunodeficiency; Congenital erythema; Infertility;	*BLM*	Various solid and hematologic malignancies
Werner Syndrome	Scleroderma-like skin; Cataract; Subcutaneous calcification; Premature arteriosclerosis; Prematurely aged facies;	*WRN*	Thyroid cancer, skin cancer, and sarcoma
Rothmund-Thomson syndrome	Poikiloderma, keratosis; Short stature; Sparse hair; Cataracts; Skeletal abnormalities;	*RECQL4*	Osteosarcoma, skin cancers
Fanconi Anemia	Bone marrow failure, physical abnormalities, organ defects	*FANCC, FANCA, FANCG, FANCF, FANCE, FANCD2, FANCL, XRCC1, SLX4, RAD51C*	Leukemia, myelodysplastic syndrome, liver cancer
Lynch syndrome	Cancer predisposition	*MLH1, MSH2, MSH6, PMS2, MLH3*	Colon cancer
Li-Fraumeni Syndrome	Cancer predisposition	*TP53*	Brain tumors, osteosarcoma, leukemia, and adrenocortical carcinoma
Breast Cancer Predisposition Syndromes	Cancer predisposition	*BRCA1, BRCA2*	Breast cancer, prostate cancer, pancreatic cancer, ovarian cancer

## Caretakers and gatekeepers in DDR

DDR proteins can be roughly classified into caretakers and gatekeepers.^65^ Caretakers protect the genome DNA by directly repairing DNA damage, while gatekeepers render the DNA repair fine-tuned with cell cycle or cell death.^66^ Caretakers and gatekeepers cooperatively maintain the genome integrity. Different types of DNA damage activate corresponding repair pathways. Of note, these DDR pathways are partially redundant, which may explain why synthetic lethal interactions are common within DDR proteins.^67^

**Fig. 2:**
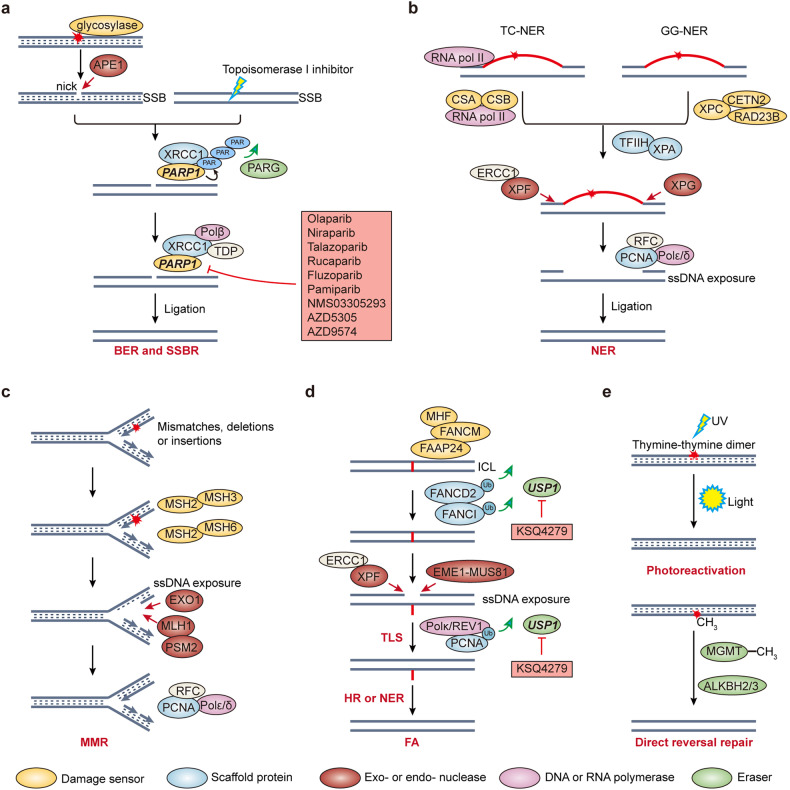
DNA repair mechanisms for damages on a single strand or interstrand crosslink. **a** BER and SSBR share the signaling/mediator proteins (such as XRCC1) and effectors (such as Polβ and TDP), while the major difference is the damage sensors. DNA glycosylases and APE1 deal with small base modifications and generate a nick (SSB). Other SSBs can be directly recognized by PARP1. PARP1 and PARG dynamically modulate PARylation level to regulate SSBR process. **b** NER deals with bulk damages that distort helix structure. These damages can be either sensed on genome by XPC-RAD23B-CETN2 complex (GG-NER), or during transcription (TC-NER) by CSA-CSB complex which bind to RNA pol II. The following processes of GG-NER and TC-NER are shared. TFIIA-XPA complex recruit endonucleases to remove distorted DNA. Then PCNA in complex with DNA polymerases are loaded to carry out gap-filling synthesis. **c** MMR corrects mismatches, insertions or deletions during replication. MSH2 heterodimerizes with MSH3 or MSH6 to form sensors of MMR. In turn MLH1-PMS2 and EXO1 cooperate to remove nucleotides including damages. Like NER, PCNA mediates the resting gap-filling synthesis. **d** ICLs could be recognized by FA core complex. The effectors of ICL repair are shared with TLS, HR or NER. USP1 mediated ubiquitination on FANCD2, FANCI or PCNA could regulate the recruitment of other repair proteins in FA or TLS, respectively. **e** Direct repair can effectively repair DNA damages by photoreactivation or MGMT, ALKBH2, and ALKBH3 mediated removal of methylated DNA damage without any help from other proteins. Ub ubiquitination; PAR poly (ADP-ribose)

Caretakers in DDR include damage sensors, signaling/mediator proteins, and effectors.^68^ As aforementioned, one of the simplest DNA damage UV-induced cyclobutane pyrimidine dimers can be repaired by light stimulated photolyase proteins (photoreactivation)^69,70^ (Fig. 2e). Small base modifications such as methylation induced by alkylating agents, oxidants and UV could cause mismatch and mutagenesis.^71^ Direct reversal repair enzymes can remove base modifications without the help of other proteins (Fig. 2e). For instance, *O*^6^-methylguanine DNA methyltransferase (MGMT) demethylates *O*^6^-methylguanine lesions through a suicide mechanism, transferring the methyl group to MGMT itself which leads to degradation.^72^ AlkB human homolog 2 and 3 (ALKBH2 and ALKBH3) directly erase methylation on *N*^1^-adenosine and *N*^3^-cytosine in a process described as “flip-out”.^73,74^ Another mechanism to tackle with small base modifications is base excision repair (BER)^75,76^ (Fig. 2a). DNA glycosylases will sense and remove the damaged base such as 8‑oxoguanine (8‑oxoG) and 5‑hydroxycytosine, leaving abasic sites or known as apurinic or apyrimidinic (AP) sites. Then AP endonuclease 1 (APE1) produce a “nick,” that is a single strand break (SSB). So the downstream effector proteins are shared between BER and SSB repair (SSBR)^77,78^ (Fig. 2a). The main difference is the sensor protein, as PARP1 recognize SSB in other conditions (for instance, induced by topoisomerase I inhibitors). The remaining BER process can be either short patch (single nucleotide replacement; the predominant way) or long patch (2 to 13 nucleotides replacement), depending on the accessibility of SSB ends. For bulk DNA adducts or crosslinks that would distort helix, nucleotide excision repair (NER) will be activated^79,80^ (Fig. 2b). Global genome NER (GG‑NER) probes the genome helix distorting lesion and transcription-coupled NER (TC‑NER) removes the lesions blocking transcription. Mismatch repair (MMR) deals with replication errors,^81,82^ including single nucleotide mismatches as well as nucleotide insertions and deletions (Fig. 2c). Like BER, both NER and MMR are also multiwise ‘cut and patch’ type reactions. Another economic but error-prone way to deal with DNA lesion is translesion synthesis (TLS)^83^ (Fig. 2d). As high fidelity repair during replication would induce breaks and replication fork collapse, TLS may help restore to duplex DNA and avoid more catastrophic consequences. Fanconi anemia (FA)^84,85^ pathway is responsible for the repair of interstrand crosslinks (ICLs) (Fig. 2d). FA core complex recognize crosslinks and recruit nucleases to incise the damaged nucleotide. In turn the effector proteins of NER, TLS or HR complete the repair.

**Fig. 3:**
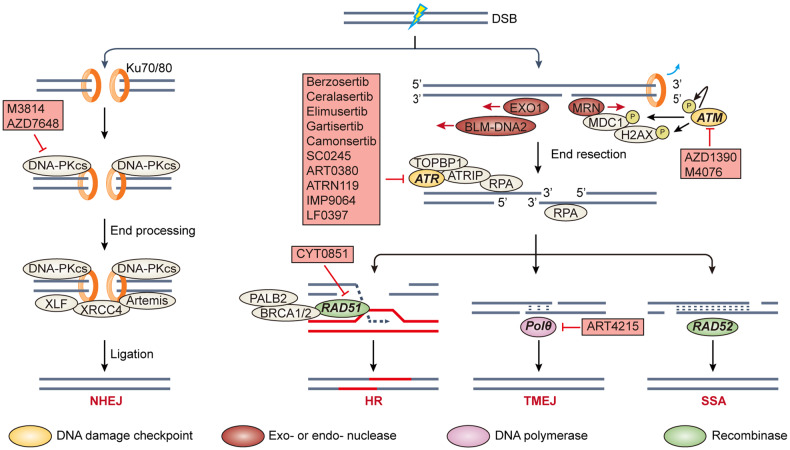
Major double strand break repair pathways. In G1 phase, the DSBs are recognized by Ku70/80 heterodimers, which recruit DNA-PKcs to form an active DNA-PK. Then DNA-PK complexes with other effector proteins to carry out end processing and ligation. After DNA replication, MRN complex may compete with Ku70/80 in the recognition of DSB ends. Endonuclease activity of MRN form a nick distant away from break point. Then 3’-5’ exonuclease MRN and 5’-3’ exonuclease EXO1 or BLM-DNA2 heterodimer carry out end resection, leaving long ssDNA exposure. MRN can also activate ATM, which phosphorylates MDC1 or γH2AX to amplify the repair signaling. Exposed ssDNA can be coated and protected by RPA, RPA interacts with ATR-ATRIP heterodimer and subsequently ATR kinase activity could be activated. RAD51 in complex with BRCA2 replaces RPA and mediates homology search for HR. If the HR process is deficient, TMEJ and SSA could compensate after end resection. Even <5 bp microhomology is sufficient for activating TMEJ, but long-range homology is required for SSA. P phosphorylation

Double strand break (DSB) is the most lethal type of DNA damage, as even one DSB could trigger cell death. 4 major DSB repair (DSBR)^86,87^ pathways have been identified (Fig. 3): homologous recombination (HR),^88^ nonhomologous end joining (NHEJ),^89^ single strand annealing (SSA)^90^ and Polθ-mediated end joining (TMEJ).^91^ NHEJ is the predominant but error-prone DSBR pathway, which could bridge DSB ends blunt or with very short overhangs. HR is error-free but only activated in G2 and M phase with the presence of homologous sister chromatin as template. As NHEJ sensor KU70/KU80 heterodimers are abundant in cells, HR sensor MRE11–RAD50–NBS1 (MRN) complex need to outcompete KU70/KU80 in the recognition of DSB ends (Fig. 3). The end resection is carried out bidirectionally from DSB ends. Eventually the long ssDNA overhangs could prevent NHEJ and facilitate HR. TMEJ recognize < 5 base pair (bp) microhomology in ssDNA overhangs after end resection^91^ (Fig. 3). Albeit error-prone, TMEJ can complete the repair when HR proteins are deficient. SSA can occur between two homologous 3′ ssDNA ends after extensive end resection (Fig. 3). In contrast, short-range end resection is sufficient to facilitate TMEJ.

**Fig. 4:**
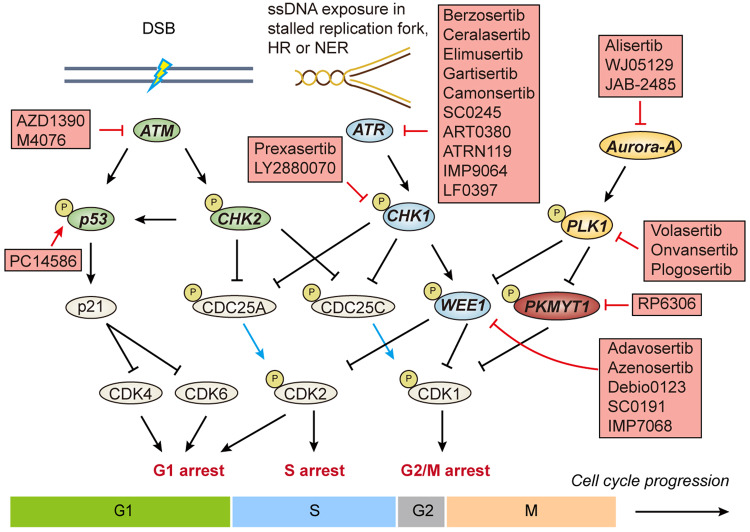
DNA damage checkpoints would be activated to by the presence of DNA damage, leading to cell cycle arrest to allow for DNA repair. ATM-CHK2-p53 axis and ATR-CHK1-WEE1 axis will be activated in response to DSBs and ssDNA exposure, respectively. PKMYT1 behaves nonredundantly from WEE1 in regulation of CDK1 activity. Aurora-A and PLK1 are implicated in mitotic entry partially through phosphorylation on WEE1 and PKMYT1 that result in their degradation. Arrows in blue denote phosphatase activity. P phosphorylation

As with gatekeepers, 3 major DNA damage checkpoints have been depicted in cells: G1/S, intra S, and G2/M checkpoint^92–94^ (Fig. 4). The cell cycle will be arrested to allow DNA repair and avoid the presence of damaged DNA in replication or mitosis. ATR-CHK1-WEE1 axis, ATM-CHK2-p53 axis, PKMYT1, and DNA-PK are the best-known DNA damage checkpoint. PLK1 and aurora kinase A (Aurora-A) are also involved in damage checkpoint regulation. Of note, ATM and ATR orchestrate both DNA damage repair and checkpoint pathways.

## PARP inhibitors

**Fig. 5:**
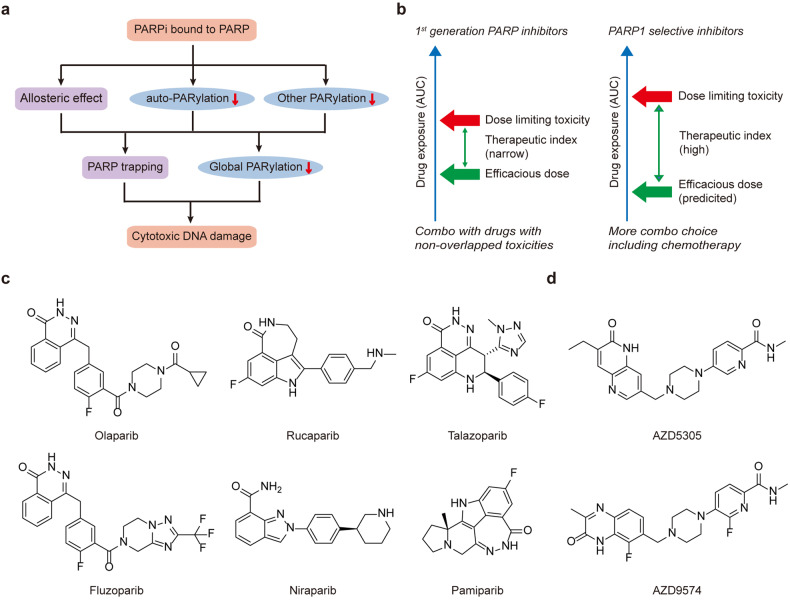
Paradigm shift in the development of PARP inhibitors. **a** Cytotoxic mechanisms of PARP inhibitors. PARP inhibitors could disrupt catalytic activity as well as cause PARP trapping on DNA, both leading to unrepaired cytotoxic DNA damage accumulation. **b** Higher therapeutic index of PARP1 selective inhibitors diversified combo opportunities. The reduced hematological toxicities of next-generation PARP1 selective inhibitors may warrant combinations with chemotherapy, while drugs with nonoverlapped toxicities may be better combo choice for first-generation PARP inhibitors. AUC: area under curve. **c** Chemical structures of 6 launched first-generation PARP inhibitors: olaparib, rucaparib, niraparib, talazoparib, fluzoparib and pamiparib. **d** PARP1 selective inhibitors AZD5305 and AZD9574

PARP1 is the prominent sensor of SSB or DSB, mediates poly-ADP-ribosylation (PARylation) on PARP1 protein itself and a subset of other substrates^95–97^ (Fig. 2a). Auto-PARylated PARP1 mediates the recruitment of X-Ray repair cross complementing 1 (XRCC1), which orchestrates the following repair process via forming complexes with other proteins.^77^ Additionally, PARP1 also involves in other repair mechanisms of NER,^98^ HR,^99^ TMEJ^100^ and other physiological processes such as chromatin remodeling,^101^ transcription,^102^ DNA replication,^103^ inflammation,^104^ metabolism,^105^ and aging.^106^ In 2005, two seminal studies demonstrated the hypersensitivity of *BRCA1* or *BRCA2* mutant cells to PARP1 inhibition,^107,108^ which paved the way for the approval of PARP1 inhibitors in patients with *BRCA* mutation. Both BRCA1 and BRCA2 are indispensable components of effective HR, so *BRCA1* or *BRCA2* mutations are strong indicators of HRD. Now all the 6 approved PARP1 inhibitors (olaparib,^24^ rucaparib,^109^ niraparib,^110^ talazoparib,^111^ pamiparib,^112^ and fluzoparib^113^) have been reckoned as first-generation inhibitors (Fig. 5c), for their dual inhibition to both PARP1 and PARP2, and even off-target activity against other PARPs or other targets.^28^ Building on the experiences of first-generation inhibitors, PARP1 selective or specific inhibitors (next-generation PARP1 inhibitors), have emerged^26^ (Fig. 5d).

### First-generation PARP inhibitors

All the approved first-generation PARP inhibitors are nicotinamide adenine dinucleotide (NAD^+^) competitive inhibitors of both PARP1 and PARP2,^14,25^ some of which even inhibit other PARP subtypes. Moreover, these inhibitors also trap PARP1/2 protein to genome DNA due to attenuated auto-PARylation of PARP1/2, which is reminiscent of topoisomerase inhibitors^114–116^ (Fig. 5a). To some extent the trapping capability of PARP inhibitors dominates over enzyme inhibition in the contribution to efficacy and toxicity^117^ (Table 4). Initially, it was hypothesized that SSBs induced by PARP inhibition would convert into DSBs that rely on BRCA1 and BRCA2 to repair. However several studies found that PARP inhibitors failed to accumulate SSBs even in *BRCA* mutant cells.^118,119^ This led to other 2 models to explain synthetic lethal mechanism between PARP1 and BRCA1/2. One is that trapped PARP would interfere with replication fork and elicit its collapse and DSBs in S phase, then HR repair is activated to resolve damages. Another model anticipates that PARP restart the stalled replication fork in a different way from HR. As with first-generation PARP inhibitors, although their enzymatic inhibition activities are comparable, the trapping activities and cytotoxic effects are significantly different.^120,121^ By mechanism, trapping activity may result from attenuated auto-PARyaltion, allosteric effect, and other reasons^122^ (Fig. 5a). Interestingly trapping abilities are inversely correlated with maximal tolerated dose (MTD) in clinic.^123,124^ For example, trapping activity of talazoparib is 100 fold more potent than olaparib (Table 4), the clinical monotherapy dose of talazoparib is 1 mg QD (once a day) whereas olaparib is 300 mg BID (twice a day).

**Table 4:** Comparison of PARP1 selective inhibitors and selected first-generation PARP inhibitors

Drugs	Olaparib	Talazoparib	NMS03305293	AZD5305	AZD9547
PARP1 IC_50_ (μM)^a^	0.007	0.009	<0.01	0.003	<0.005
PARP2 IC_50_ (μM)^a^	0.006	0.030	0.691	>1.4	>93
PARP1/2 selectivity fold	1	3	>200	>460	19107
PARP3/5a/6 IC_50_ (μM)^a^	0.2/70/1.8	0.2/1.9.1.1	0.5/>10/not reported	3.4/ > 89/26	All > 100
PARP1 trapping^b^	+	++	-	++	Yes
PARP2 trapping^b^	+	++	-	-	-
DLD1 *BRCA2*^−/−^ cell line antiprolifertive GI_50_ (nM)	11	0.5	Not reported	0.4	1.4
Pgp substrate	Yes	Yes	No	Yes	No
CNS penetration	Limited, Rat/Monkey Kpuu: <0.03/<0.1	Limited, Rat Kpuu: <0.03	Yes, with a brain/plasma ratio of 4-10 in rats and mouse	Limited, Rat/Monkey Kpuu: <0.05/<0.01	Yes, with Rat Kpuu 0.31

^a^Determined by a fluorescence polarization assay

^b^Determined by an immunofluorescence–based assay

PARP inhibitors have been approved for the treatment of ovarian cancer, HER2-negative breast cancer, pancreatic ductal adenocarcinoma (PDAC) and metastatic castration-resistant prostate cancer (mCRPC) (Table 1). Recently, olaparib, rucuparib and niraparib voluntarily withdraw the indication of late-line treatment therapy for ovarian cancer patients, due to the potential detrimental effect on patient overall survival. The approvals of PARP inhibitors for the treatment of late line ovarian cancer were largely based on objective response rate (ORR) and median duration of response (mDOR), and median overall survival (mOS) data have not yet matured at that time.^125–127^ Of note, median progression free survival (mPFS) but not mOS are primary endpoints for most clinical trials of PARP inhibitors, which have supported other approvals in ovarian cancer. However, the recent updates of PARP inhibitors as maintenance therapy seem more promising.^128–132^ For example, in *BRCA* mutant ovarian cancer patients responsive to first-line platinum-based chemotherapy,^133^ olaparib maintenance therapy prolonged both mPFS (56.0 vs 13.8 months, hazard ratio: 0.33) and mOS (not reached vs 75.2 months, hazard ratio: 0.55) than placebo control.

The biomarkers for patient selection in early approvals were predominately *BRCA* mutations (Table 1). Subsequently, HRD including not only *BRCA* but other HR repair gene deficiencies could benefit from PARP inhibitors in ovarian cancer or prostate cancer.^130,134–136^ Furthermore, in platinum sensitive ovarian cancer patients, olaparib, rucaparib and niraparib extended mPFS irrespective of *BRCA* or HR repair gene background. Maybe platinum-sensitive patients harbor other vulnerable gene signatures beyond *BRCA* mutations and HRD. Now first-generation PARP inhibitors are still widely exploited in clinic for more indications, novel combinations and biomarkers.

### Next generation PARP1 selective inhibitors

Albeit with their impressive efficacies in clinic, hematological toxicities such as anemia, neutropenia, and thrombocytopenia are common adverse events (AEs) during first-generation PARP inhibitors treatment.^137^ These safety risks lead to dose discontinuation and reduce combination possibilities with chemotherapy or other kinds of therapies. The discovery of next generation PARP1 selective inhibitors can be attributed to fundamental mechanistic understandings of different PARP proteins. Several studies point out that PARP2 is linked with hematological toxicities,^138,139^ but PARP1, the primary responder in DDR, is predominately required for efficacy. Double knockout of both PARP1 and PARP2 would impair normal embryonic development.^140^ Moreover, PARP5A/B inhibition are believed to be responsible for gastrointestinal adverse effects.^141^ In this sense a PARP1 selective inhibitor may reduce the toxicity whilst maintain the antitumor activity, thus leaving a higher therapeutic index for more combination choice (Fig. 5b).

NMS-03305293 (also known as NMS-P293), the first claimed PARP1 selective inhibitor proceeded to clinical investigation, is developed as a potent PARP1 enzyme inhibitor but not a trapper (Table 4).^142^ NMS-03305293 selectively suppresses the HR deficient cell growth in vitro and in vivo, accompanied by the significantly PAR reduction. Strikingly, NMS-03305293 could penetrate the blood brain barrier and shows synergistic effect when combined with temozolomide (TMZ) in glioblastoma (GBM) tumor models.^143^ Now NMS-03305293 is under clinical investigation by Nerviano Medical Sciences in collaboration with Merck (Table 2).

The second PARP1 selective inhibitor in clinic, AZD5305, displays over 400 fold selectivity to PARP2 in a fluorescence polarization competition assay (Table 4 and Fig. 5d).^117^ Different than NMS-03305293, AZD5305 is also a strong PARP1 trapper.^144^ In a well-established cell-based trapping assay, AZD5305 selectively trap PARP1 at nanomolar range, which was much more potent than olaparib. Strikingly AZD5305 failed to trap PARP2 even at micromolar concentration. With optimized pharmacokinetics, AZD5305 caused tumor regression at 100 fold lower dosage than olaparib. In addition, AZD5305 retained the selective killing to *BRCA* deficient cell line both in vitro and in vivo, when compared to *BRCA* wild type isogenic cells. In rat toxicity studies, AZD5305 demonstrated minimal reduction with respect to reticulocytes, erythroids, neutrohils and platelets, which was ascribed to the avoidance of PARP2 inhibition and less promiscuity in secondary pharmacology.^145^ Preliminary data from first-in-human clinical trial (PETRA) showed that AZD5305 outcompeted first-generation PARP inhibitors in safety profile.^146^ As reported, AZD5305 dosage has been escalated to 140 mg daily, with only 3% patients require a dose reduction due to AEs, versus 25% - 53% in patients receiving a full dose of first-generation PARP inhibitors. In addition, AZD5305 has achieved higher steady state C_trough_/target effective concentration (TEC) fold than first-generation PARP inhibitors, even at the starting dose 10 mg daily (C_trough_ /TEC: 7.12). Remarkably, patients resistant to prior PARP inhibitor treatment also responded to AZD5305. All these findings warrant a wide therapeutic index of AZD5305 and more combination opportunities in clinic (Table 2).

Recently, AZD9574, another PARP1 selective inhibitor with improved brain penetrant property, initiated first-in-human clinical trial. AZD9574 retained the selectivity and potency of AZD5305 (Table 4 and Fig. 5d), and dramatically regressed tumor growth in both subcutaneous and intracranial models.^147^ With low P-glycoprotein (P-gp)/breast cancer resistance protein (BCRP) driven efflux, AZD9574 displayed higher Kpuu in both rat (0.31) and monkey (0.79). In parallel, the rat Kpuu of first-generation PARP inhibitors were all < 0.1 and for AZD5305 was < 0.05.^148,149^ Hence AZD9574 can be explicitly differentiated from other PARP inhibitors as the first brain penetrant PARP1 selective inhibitor and trapper in clinic (Table 2).

## DDR gatekeepers as antitumor targets

### ATR inhibitors

In human, *ATR* gene is essential in development and its deficiency resulted in a rare autosomal recessive disorder called Seckel syndrome (Table 3), which is featured by intrauterine growth retardation, microcephaly, and developmental defects.^150^ ATR kinase belongs to the phosphatidylinositol 3-kinase-related kinase (PIKK) family and functions as the apical responder to ssDNA exposure.^151^ ssDNAs are abundant in numerous physiological processes including DNA replication, HR, NER, and cancer cells with replication stress. ssDNA-bound RPA recruits ATR in complex with ATR interaction protein (ATRIP) to the sites of replication stress or DNA damage. Upon the loading of Rad9–Rad1–Hus1 (9–1–1) complex to these sites, DNA topoisomerase 2-binding protein 1 (TOPBP1) will be recruited and serves as an allosteric activator of ATR/ATRIP complex (Fig. 3). The activated ATR/ATRIP mediates the phosphorylation of a broad range of substrates involved in DNA repair, control of replication firing, restart of stalled replication fork and cell cycle arrest.^152^ p53, CHK1, BRCA1, WRN and minichromosome maintenance 2 (MCM2) are among the best-known ATR substrates. The activated CHK1 catalyzes the inhibitory phosphorylation on CDC25 phosphatases and stimulatory phosphorylation on WEE1, which converge on the prevention of CDK1 activation and lead to cell cycle arrest^152^ (Fig. 4). MCM2 in complex with MCM7 forms a helicase that unwinds the DNA duplex during replication.^16^ It is suggested that ATR was indispensable for regulating replication in both normal tissues and cancer cells. Concerning cancer cells suffering from replication stress, it may confer a window for pharmacological ATR inhibition.^153^

**Fig. 6:**
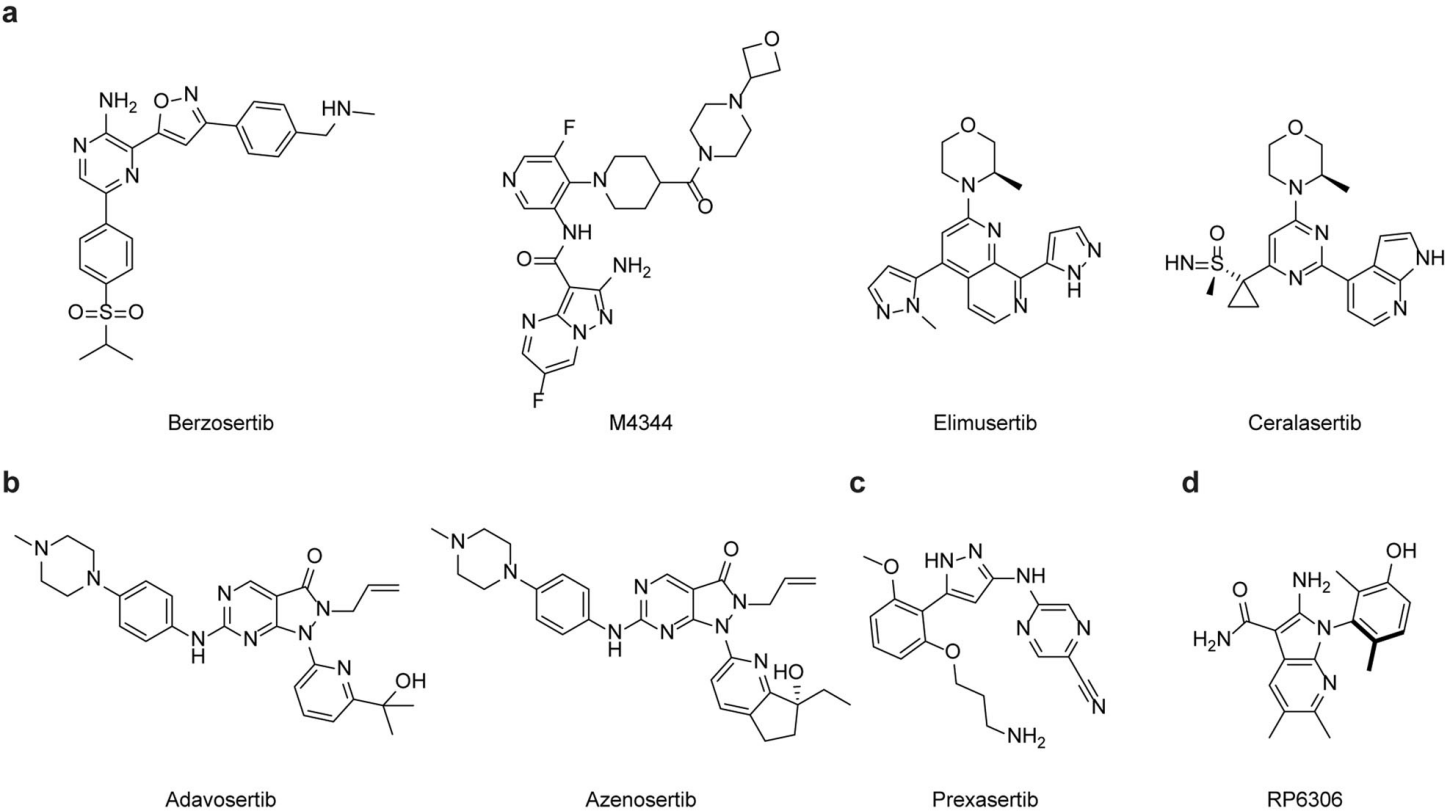
Chemical structures of selected DNA damage checkpoint inhibitors. **a** ATR inhibitors berzosertib, M4344, elimusertib and ceralasertib. **b** WEE1 inhibitors adavosertib and azenosertib. **c** CHK1/2 inhibitor prexasertib. **d** PKMYT1 inhibitor RP6306

Most clinical-stage ATR inhibitors, such as berzosertib, ceralasertib, elimusertib, gartisertib, and camonsertib are all ATP-competitive with highly selectivity over other PIKK members or other kinases, whereas their potencies on ATR are different (Table 5 and Fig. 6a).^154^ These ATR inhibitors accumulated DNA damage, demonstrated hypersensitivity in *ATM* mutant cancer cell lines and synergies with radiation, chemotherapy or PARP inhibitors in CDX (cell line derived xenograft) and PDX (patient-derived xenograft) models.^155–158^ These preclinical findings are consistent with ATR function in the maintenance of genome integrity, and overlapped downstream effectors of ATR and ATM may imply a synthetic lethal relationship. Furthermore, ATR inhibition can facilitate antitumor microenvironment by reducing PDL1 expression, promoting CD3^+^ or NK infiltration and activation of nucleic acid sensing pathway.^159,160^ Thus ATR inhibitors also showed synergistic effect with immune-oncology therapeutics such as anti-PD(L)1 antibodies.^161^ Interestingly given the differences in ATR potency and physicochemical property, the safety profile of ATR inhibitors as monotherapy in patients illustrated somewhat similarities. Hematological toxicities including anemia, neutropenia, and thrombocytopenia were common in ceralasertib, elimusertib, or camonsertib monotherapy.^162–164^ One exception is berzosertib, which is intravenously administrated once or twice a week.^165^ No dose-limiting toxicities (DLTs) were observed during berzosertib dose escalation, and the monotherapy recommended phase 2 dosage (RP2D) was determined at 240 mg/m^2^ due to the limit of infusion volumes. However, relatively lower patient compliance due to intravenous dosing route may impede the possibility for more intensive schedule for berzosertib.

**Table 5:** Comparison of selected clinical-stage ATR inhibitors as monotherapy

Drugs	Berzosertib	Ceralasertib	Elimusertib	Camonsertib
In vitro activity				
ATR IC_50_ (nM)	0.17^a^	4	7	1
Selectivity fold to other PIKK kinases	All >100	All >300	ATM > 200 DNA-PK > 40 PI3K > 400 mTOR > 6 mTOR^c^ > 60	mTOR^b^ > 120 ATM^b^, DNAPK^b^, and PI3K^b^ >2000
LoVo^c^ antiprolifertive IC_50_ or GI_50_ (μM)	Not reported	0.44	0.071	0.028
Monotherapy behavior in human				
Dosing route	Intravenous	Oral	Oral	Oral
MTD	Not reached, RP2D was 240 mg/m^2^, once- or twice-weekly	160 mg BID	40 mg BID, 3 days on/4 days off	160 mg QD, 3 days on/4 days off
DLTs or SAE	no DLTs observed	thrombocytopenia, pancytopenia and elevated amylase	anemia, neutropenia, thrombocytopenia, fatigue, nausea	anemia, neutropenia, thrombocytopenia

^a^*K*_*i*_ value

^b^Selectivity fold at cellular level

^c^An *MRE11* mutant cell line frequently used in ATR inhibitor activity evaluation

Berzosertib (previously known as M6620 or VX970 or VE822, developed by Vertex and Merck) was the first ATR inhibitor entering clinical investigations^166^ (Fig. 6a). The preliminary clinical data of berzosertib in combination with chemotherapy have been extensively reported. By combination with cisplatin,^165^ partial response was observed in 4 out of 31 patients who experienced disease progression following standard therapy. In later-line nonsmall cell lung cancer (NSCLC) patients,^167^ berzosertib combined with gemcitabine led to an ORR at 10.5% (90% confidence interval (CI), 3.7 – 22.5%) and DCR (disease control rate) at 68.4% (90% CI, 53.9 – 80.7%), respectively. Of note, patients with high tumor mutation burden (TMB) or loss of heterozygosity (LOH) score tended to be more responsive to berzosertib and gemcitabine co-treatment. In platinum-resistant high-grade serous ovarian cancer (HGSOC) (Table 6),^168^ berzosertib plus gemcitabine significantly prolonged mPFS compared to gemcitabine alone (22.9 vs 14.7 weeks, hazard ratio 0.57, one-sided log-rank test *p*=0·044). Of note, the safety profiles were comparable in combination group and gemcitabine monotherapy. However, the ORR was lower in combination group, which is uncommon. In the second line small cell lung cancer (SCLC) patients,^169^ the addition of berzosertib to standard chemotherapy topotecan achieved partial response in 9 out of 25 patients. 17/25 patients experienced tumor regressions. Strikingly, most major AEs can be attributed to topotecan, but not the combination. Now berzosertib is still under active clinical explorations by combination with chemotherapy, radiation, PARP inhibitor or anti-PD(L)1 antibodies (Table 2).

**Table 6:** Preliminary clinical data of DDR targeting agents in platinum resistant or refractory ovarian cancer

NCT	NCT02595892	NCT04497116	NCT01164995	NCT02151292	NCT03579316^a^	NCT04516447	NCT02203513	NCT02632448
Drugs	Berzosertib + Gemcitabine vs Gemcitabine	Camonsertib	Adavosertib+ Carboplatin	Adavosertib + Gemcitabine vs Gemcitabine	Adavosertib vs Adavosertib + Olaparib	Azenosertib+chemotherapy	Prexasertib	LY2880070+Gemcitabine
No. of patients	34 vs 36	20	23	61 vs 33	35 vs 35	56	49	27
Confirmed % of *BRCA* mutant or HRD	18% vs 14%	85%	9% *BRCA1* mutation	16% vs 12%	48% *BRCA* mutation	Not reported	*BRCA* wildtype	Not reported
% of prior PARPi treatment	32% vs 19%	90%	Not reported	Not reported	100%	14%	46%	Not reported
% of prior bevacizumab treatment	29% vs 25%^b^	Not reported	4%	Not reported	Not reported	46%	81%	Not reported
ORR (%)	3% vs 11%	1 CR, 3 PRs, 1 CA125 response	43% (95% CI, 22% - 66%)	23% vs 6%	23% (90% CI, 12% – 38%) vs 29% (16% – 44%)	Total 30.2%; ZnC3+paclitaxel: 62.5%; ZnC3+carboplatin: 45.5% ZnC3+PLD^c^: 12.5%	30.7%	7.4%
mPFS	22.9 weeks (90% CI 17.9–72.0) vs 14.7 weeks (9.7–36.7), hazard ratio 0.57, 0.33–0.98; one-sided log-rank test *p* = 0.044	Not reported	5.3 months (95% CI, 2.3 to 9.0 months)	4.6 months (95% CI 3.6–6.4) vs 3.0 months (1.8–3.8), hazard ratio 0.55 [95% CI 0.35–0.90]; log-rank *p* = 0_·_015	5.5 months (90% CI, 3.9–6.9) vs 6.8 months (4.3–8.3)	Not reported	5.8 months (range 1.7-26.4 months).	Not reported
mOS	59.4 weeks (90% CI 33.7–84.4) vs 43.0 weeks (34.4–67.9) hazard ratio 0.84, 0.53–1.32; one-sided log-rank test *p* = 0.26	Not reported	12.6 months (95% CI, 4.9 to 19.7),	11.4 months (95% CI 8.2–16.5) vs 7.2 months (5.2–13.2); hazard ratio 0.56 [95% CI 0.35–0.91]; log-rank *p* = 0.017	Not reported	Not reported	Not reported	Not reported

^a^A two-arm noncomparative trial

^b^Previous antiangiogenic therapy

^c^PLD: Pegylated liposomal doxorubicin

In addition to berzosertib, Vertex and Merck also developed an oral ATR inhibitor gartisertib (also known as M4344) in clinic (Fig. 6a). Gartisertib seemed to be more potent than berzosertib at cellular level.^158^ Interestingly, cancer cell lines with replication stress (RepStress) and neuroendocrine (NE) gene expression signatures were hypersensitive to gartisertib. RepStress and NE gene expression signatures are of candidate predictive biomarkers to stratify patients for ATR inhibitors.

Ceralasertib (also known as AZD6738, developed by AstraZeneca) is the first oral ATR inhibitor in clinic (Fig. 6a). Ceralasertib was optimized from a lead compound AZ20, with improved solubility and avoidance of CYP3A4 time dependent inhibition.^156^ Albeit ceralasertib demonstrates efficacy as single agent, the clinical development is centered on combination. For example, in melanoma patients resistant to prior anti-PD1 therapy,^170^ ceralasertib (dose escalation from 40 mg QD to 240 mg BID) in combination with paclitaxel delivered ORR and DCR at 33.3% (95% CI, 18.0–51.8) and 60.6% (95% CI, 42.1%–77.1%), mPFS and mOS at 3.6 (95% CI, 2.0–5.8) and 7.4 (95% CI, 5.7–11.9) months, respectively. The RP2D was determined at 240 mg BID days 1-14 every 28 days. Interestingly, in another trial treating melanoma patients resistant to prior anti-PD1 therapy,^171^ ceralasertib at fixed dosage (240 mg BID days 15-28 every 28 days) combined with anti-PDL1 antibody durvalumab generated ORR and DCR at 31.0% and 63.3%, mPFS and mOS at 7.1 (95% CI, 3.6-10.6) and 14.2 (95% CI, 9.3-19.1) months, respectively. Seemingly that the addition of durvalumab to ceralasertib improved the duration of clinical activity than paclitaxel. In advanced gastric cancer (AGS),^172^ co-treatment of ceralasertib and durvalumab also brought benefit. Of note, AGS patients with loss of ATM expression or HRD benefited more than those with intact ATM or low HR signature. By combination with olaparib, ceralasertib also showed preliminary response in HGSOC,^173^ SCLC^174^ and breast cancer.^175^ Impressively, 6 out of 13 PARP inhibitor resistant HGSOC patients demonstrated partial response upon co-treatment of olaparib and ceralasertib, indicating that ATR inhibition could circumvent PARP inhibitor resistance.^173^ Recently, ceralasertib in combination with durvalumab initiates a phase 3 study for the treatment of later line NSCLC patients (Table 2).

Elimusertib (also known as BAY1895344, developed by Bayer) was more potent than ceralasertib and berzosertib at cellular level, meanwhile was comparable to M4344^158,176^ (Fig. 6a). In a CDX model, elimusertib monotherapy outcompeted ceralasertib and berzosertib at their MTD dosages, due to its longer and sufficient exposure above antiproliferative IC_50_. At the MTD dosage in human (40 mg BID 3 days on/4 days off),^163,177^ elimusertib brought preliminary single agent benefit to patients in clinic, but only 5 out 143 patients achieved PR. In patients with ATM loss, the ORR was slightly increased to 9% and DCR was 65%. Of note, a less intensive dosing schedule 3 days on/11 days off may help mitigate toxicities. Interestingly, two intermittent strategy, 40 mg/kg, BID, 3 days on/4 days off and 60 mg/kg, BID, 3 days on/11 days off generated comparable efficacy in several CDX models. So a less intensive schedule with enhanced dosage may not sacrifice efficacy and can improve tolerability.^178^ Now elimusertib is under investigation by combination with chemotherapy, niraparib, or anti-PD1 antibody pembrolizumab (Table 2). And the dosing schedule of elimusertib in the combination scenario may need more explorations.

Likewise, another oral ATR inhibitor camonsertib (also known as RP3500, developed by Repare) reported single-agent activity in clinic.^179^ The RP2D of camonsertib monotherapy was determined at MTD of 160 mg QD, 3 days on/4 days off.^164^ In platinum drugs or PARP inhibitors pretreated ovarian cancer, 5 out of 20 benefited from camonsertib monotherapy, including 1 complete response, 3 PRs and 1 CA125 reduction (Table 6). The ORRs were modest in patients harboring *ATM* (12%) or *BRCA* (14%) deficiency. For the combination scenario, camonsertib and PARP inhibitors dosed concomitantly 3 days on/4 days off outperformed sequential (PARPi for 3 days followed by camonsertib for 3 days, then 1 day off) in preclinical evaluations. And shortened duration of drug exposure help ameliorate tolerability with minimal effect on red blood cell and reticulocyte.^180^ Now camonsertib is co-developed by Repare and Roche.

### WEE1 inhibitors

WEE1 kinase catalyzes the inhibitory phosphorylation of CDK1 and CDK2 on conservative Tyr15, thereby acting as a G2/M checkpoint and a guardian for DNA replication.^181,182^ With the presence of DNA damage or uncompleted DNA replication, ATR-CHK1 axis phosphorylates and stimulates WEE1, which in turn inactivates CDK1 to avoid premature mitosis (Fig. 4). Otherwise to override G2/M checkpoint, PLK1-mediated WEE1 phosphorylation promotes WEE1 degradation via the ubiquitin ligase complex. During S phase, WEE1 is implicated in the maintenance of genome integrity through CDK2-regulated replication initiation and Mus81-Eme1 endonuclease mediated processing of stalled replication forks.^183,184^ In light of its sophisticated functions, *WEE1* depletion or inhibition render cancer cells die of replicative or mitotic catastrophe, and hypersensitive to chemotherapy or radiation as expected.^185,186^

Adavosertib (also known as AZD1775 or MK1775) is the first ATP-competitive WEE1 inhibitor in clinic (Fig. 6b). In preclinical animal studies, adavosertib showed antitumor effect either as single agent or a sensitizer to chemotherapy such as gemcitabine, 5-fluorouracil and platinum drugs.^186–188^ In vitro, the combination with chemotherapy resulted in premature mitosis and mitotic catastrophe, whereas single agent activity of adavosertib was more related to DNA damage accumulation in S phase and replicative catastrophe. Albeit cancer cells with G1 checkpoint dysregulation are believed to be more reliant on G2/M checkpoint to maintain homeostasis, the correlation of adavosertib sensitivity and *TP53* status appeared to be controversial.^189^ Of note, an unbiased mass spectrometry (MS)-based chemical proteomics survey uncovered a set of kinases hit by adavosertib, and adavosertib was equally potent against WEE1 and PLK1.^190^ Severe AEs especially gastrointestinal toxicities and myelosuppression were observed in patients receiving adavosertib.^191,192^ Although these AEs were broadly identified in DDR targeting agents, the contributions of off-target kinase inhibition cannot be neglected. Limited by therapeutic index in clinic, the optimal dosage and schedule for adavosertib as monotherapy or in combination with chemotherapy were both determined in unconventional intermittent manners. Even with these challenges, adavosertib achieved preliminary response in various cancer conditions. In patients with recurrent uterine serous carcinoma (USC),^193^ adavosertib monotherapy brought about an ORR of 29.4% (95%CI 15.1-47.5%), and mPFS and mDOR were determined at 6.1 and 9.0 months, respectively. In high-grade serous ovarian cancer patients that were refractory to or relapse after platinum drugs treatment,^194^ adavosertib plus gemecitabine extended both mPFS (4.6 vs 3.0 months, hazard ratio 0.55, log-rank *p* = 0.015) and mOS (11.4 vs 7.2 months, hazard ratio 0·56, log-rank *p* = 0.017) compared to gemcitabine alone (Table 6). Also in late line ovarian cancer patients who progressed on PARP inhibitor treatment,^195^ adavosertib monotherapy induced an ORR of 23% (90% CI, 12%-38%), a clinical benefit rate (CBR) of 63% (90% CI, 48%-76%), and mPFS of 5.5 (90% CI, 3.9–6.9) months (Table 6). In another noncomparative arm, adavosertib and olaparib co-treatment delivered an ORR of 29% (90% CI, 14%-44%), a CBR of 89% (90% CI, 76%-96%) and mPFS of 6.8 (90% CI, 4.3–8.3) months. The benefit was achieved irrespective of *BRCA* background, however, grade 3/4 adverse effects were common in both arms. In locally advanced pancreatic cancer patients,^196^ adavosertib in combination with gemcitabine and radiation extended mPFS and mOS to 9.4 and 21.4 months respectively, both longer than historical results. Recently, adavosertib was deprioritized by AstraZeneca.

In light of adavosertib experiences, two more selective ATP-competitive WEE1 inhibitors azenosertib (also known as ZnC3, developed by Zentalis)^197^ and Debio0123 (developed by Debio)^198^ were under clinical investigations. Although structurally analogous to adavosertib (Fig. 6b), azenosertib was obviously less promiscuous in a panel of kinases. Strikingly, the higher selectivity of azenosertib left a safer AE profile in clinic when compared to adavosertib at similar dosage of 300 mg QD.^199,200^ Moreover, azenosertib can be dosed continuously while adavosertib had to be intermitted due to safety issues. Likewise, azenosertib obtained preliminary response in USC patients as monotherapy or platinum-resistant ovarian cancer by combination with chemotherapy (Table 6). Recently, azenosertib was shown to be more sensitive in cyclin E1 overexpression ovarian cancer cell line in vitro and in vivo. Cyclin E1 overexpression via *CCNE1* amplification or independent mechanisms is quite common in HGSOC patients, which may be employed for responder enrichment in clinic.^201^ Now Zentalis teams up with Pfizer and GlaxoSmithKline (GSK) for the development of azenosertib (Table 2).

The structure of Debio0123 remains undisclosed. Compared to adavosertib, Debio0123 curtailed the activity against PLK1,^202^ which may ameliorate tolerability. In a dose-escalation phase 1 trial, target engagement in patients has been confirmed by using skin tissue pCDK1 reduction as a surrogate.^203^ More patient data have not yet come with respect to safety profile and response of Debio0123. Recently, it is reported that Debio0123 can penetrant blood-brain barrier (BBB) with mean brain-to-plasma concentration ratios of ~0.6 and 1.52 and 4 in mice, rats, and monkeys, respectively.^204^ Of note, Debio0123 monotherapy or in combination with TMZ produced remarkable efficacy in orthotopic GBM models. A new clinical trial of Debio0123 in combination with TMZ and radiation has just initiated for the treatment of GBM (Table 2).

### ATM inhibitors

ATM, another member of PIKK family, plays an integral role in DSB response.^205^ Mutations in *ATM* gene are associated with a hereditary genomic instability disorder called ataxia-telangiectasia (Table 3), which is featured by progressive ataxia, telangiectasias, weakened immune system, and hypersensitivity to ionizing radiation.^206,207^ ATM could be recruited to DSB sites by MRN complex, in turn mediates the phosphorylation of a subset of substrates such as Serine 139 on histone H2AX (referred as γH2AX) and mediator of DNA damage checkpoint 1 (MDC1) to orchestrate DSB response network^208^ (Fig. 3). Of note, ATM and its well-documented substrate CHK2 both phosphorylated p53, leads to p53 stabilization and G1/S checkpoint activation (Fig. 4). In S phase, activated ATM-CHK2 axis induces phosphorylation and degradation of phosphatase CDC25A. CDC25A is responsible for the removal of inhibitory phosphorylation of CDK2, which is required for DNA replication. Albeit regarded as the most lethal type of DNA damage, DSB is scarce in normal physiological conditions.^209^ This may explain why ATM was not as essential as ATR to normal cell.

Historically KuDOS Pharma (acquired by AstraZeneca) reported a series of small molecule ATM inhibitors.^205^ These ATM inhibitors failed to cause cytotoxic effect as single agent, but sensitized cancer cells to DSB inducers such as radiation and topoisomerase inhibitors. In this way, ATM inhibitors should be developed for combination scenarios. Consistent with ATM biological function, ATM inhibitors cannot potentiate DNA alkylating agents, platinum drugs and taxanes in vitro. AZD0156 (developed by AstraZeneca) is the first ATM inhibitor entering clinical evaluations. AZD0156 was of high potency and showed remarkable selectivity over other PIKK family kinases.^210^ AZD0156 abrogated the DSB repair signaling induced by IR in vitro and showed synergistic effect when combined with IR or isomerase inhibitors in vivo. Of note, AZD0156 also potentiate PARP inhibitor olaparib in PDX models.^211^ Combination of AZD0156 and olaparib led to enhanced accumulation of cells arrested in G2/M phase and triggered more apoptosis. However severe AEs especially hematological toxicities emerged in patients treated with AZD0156 and olaparib combination.^212^ We anticipated that systemic administration of AZD0156 and olaparib exacerbated on-target toxicities in blood. Now AZD0156 has been removed from AstraZeneca pipeline.

Other 2 potent and selective ATM inhibitors AZD1390 and M4076 are now under clinical investigations. Compared to AZD0156, AZD1390 demonstrated brain-penetrant capability in both cynomolgus monkey (K_puu_ = 0.33)^213^ and healthy human (K_puu_ = 0.24 mL*cm^−3^, determined by positron emission tomography using radiolabeled AZD1390).^214^ In mouse intracranial xenograft models, oral administrated AZD1390 dramatically extended survival by combination with radiation and temozolomide. Of interest, glioma cell line screen indicated that cells harboring *TP53* mutation were more sensitive to AZD1390 and radiation combination compared to *TP53* wildtype.^213^ This may be attributed to S phase accumulation of *TP53* mutant glioma cell lines, which render cells more reliant on HR to repair radiation-induced DSBs. M4076 also displayed synergistic effect when combined with radiation, topoisomerase, and PARP inhibitors in preclinical models, albeit the BBB permeability of M4076 was not reported.^215^ A recent study illustrated that residual cancer cells which survive oncogene-targeted therapies developed synthetically dependency on ATM, and combination of AZD0156 and osimertinib (a 3^rd^ generation EGFR inhibitor) generated synergistic effect and eradicate residual cancer cells in vivo.^216^ This may broaden the combination opportunities for ATM inhibitor in clinic.

### DNA-PK inhibitors

DNA-PK is the major signaling/mediator protein in NHEJ, the error-prone but default DSBR pathway for cells outside S or G2 phase^217,218^ (Fig. 3). As a member of PIKK kinase family, DNA-PK enzyme consists of a catalytic subunit (DNA-PKcs) and a regulatory heterodimer Ku (Ku70/Ku80). Ku70/Ku80 heterodimers are abundant in cells, so as to instantly recognize and localize DSB ends which are blunt or with very short ssDNA overhangs. DNA-PKcs is then recruited to the heterodimer to form an active DNA-PK complex (Fig. 3). DNA-PK serves as a scaffold for loading other NHEJ effector proteins, which will complete end processing and ligation processes. Besides, DNA-PKcs involves in other cellular processes such as replication stress response,^219^ transcription,^220^ telomere maintenance & capping^221,222^ and innate immunity.^223^ Strikingly, deficiencies in DNA-PKcs encoding gene *PRKDC* dampen T and B cell development and lead to severe combined immunodeficiency (SCID) in mice.^224^ With its versatile roles in physiological processes, DNA-PKcs may be essential to certain normal tissues.

Targeting DNA-PKcs by siRNA or pharmacological inhibition leads to potentiation of cancer cells to radiation and chemotherapy.^225,226^ This finding evoked a great deal of interest in DNA-PKcs inhibitor development, some of which have advanced into clinical investigation.^227^ Unfortunately, most DNA-PKcs inhibitors have been deprioritized from clinical development, including DNA-PKcs selective inhibitors M3814^228^ and AZD7648.^229^ Both M3814 and AZD7648 are ATP-competitive inhibitors, and demonstrated selectivity over other PIKK kinases. As expected, M3814 or AZD7648 potentiated radiation and chemotherapy both in vitro and in vivo. AZD7648 was also explored in combination studies with olaparib in cells with *ATM* deficiency, as *ATM* deficiency may cause synthetic lethality with DNA-PKcs inhibition. Preliminary clinical data indicated that M3814 was well tolerated as monotherapy, accompanied by limited patient response.^230^ By combination with radiation, the tolerated dose was lowered for M3814, even though preliminary efficacy was observed.^231^ With limited information, we cannot precisely rule out the underlying reasons for the discontinuation of M3814 and AZD7648. But the unsatisfactory patient responses and potential competition with ATM inhibitors M4076 and AZD1390 when combined with radiation should be taken into consideration.

### CHK1/2 inhibitors

Cell cycle checkpoint CHK1 and CHK2 are key downstream regulators of ATR and ATM, respectively^232,233^ (Fig. 4). Albeit ATR-CHK1 axis and ATM-CHK2 axis aforementioned are activated by different conditions, substrates and signaling circuities of CHK1 and CHK2 are partially overlapped. With respect to DDR-associated cell cycle regulation, CHK2 is in principle implicated in G1 checkpoint whereas CHK1 is mainly activated in intra S and G2/M checkpoint. *CHK1* knockout in mice resulted in early embryonic lethality, in contrast *CHK2* knockout mice developed normally, which implies that CHK1 is more essential than CHK2.^232^ Providing ATR inhibitors are hypersensitive in *ATM-*deficient conditions, yet the synthetic lethal relationship between CHK1 and CHK2 remain elusive. Based on the extent of CHK2 potency, most clinical-stage CHK1 inhibitors can be classified into CHK1-selective (for example rabusertib and SRA737) or CHK1/2 dual inhibitors (for example AZD7762, PF-477736 and prexasertib).^234^ Albeit entering clinical investigations for more than a decade, there has been a long track records of deprioritization in the development of CHK1 inhibitors, irrespective of their CHK1/2 selectivity. Notably, prexasertib (Fig. 6c) and LY2880070 are still under active development (Table 2).

As an ATP-competitive inhibitor, prexasertib (also known as ACR-368 or LY2606368) potently inhibited CHK1, and CHK2 to a lesser extent.^235^ Prexasertib treatment induced replication catastrophe, premature mitosis and apoptosis in cancer cells. In vivo prexasertib inhibited the growth of tumor models with various histological backgrounds and potentiate chemotherapy and PARP inhibitors.^236–239^ The intravenous dose of prexasertib in clinic was determined at MTD, 105 mg/m^2^ once every 14 days.^240^ The most common treatment-emergent adverse event (TEAE) was grade 4 neutropenia, typically lasting <5 days. In heavily pretreated platinum-resistant high-grade serous ovarian cancer (HGSOC) patients (Table 6),^241^ monotherapy of prexasertib brought an ORR of 30.7%, and the clinical benefit rate (PR+CR+SD >4 months) was determined at 84.6%. The mPFS and mDOR among PRs were 5.8 and 5.5 months, respectively. As an intravenous and less selective CHK1 inhibitor, only one dose in each cycle may considerably balance compliance, safety and efficacy for prexasertib. Providing that the T_1/2_ of prexasertib was around 11 - 12 hours, the duration of exposure at RP2D is shortened compared to other DNA damage associated cell cycle checkpoint inhibitors dosing more intensively. Now prexasertib is developed by Acrivon Therapeutics, that employed a diagnosis test for the stratification of patients sensitive to prexasertib.

LY2880070 (also known as ESP-001) is claimed as an oral and selective ATP-competitive CHK1 inhibitor, however to our knowledge preclinical data of LY2880070 is still unavailable. The dosing escalation study of LY2880070 monotherapy compared QD and BID dosing days 1 - 5 in every 21-day cycle in patients.^242^ Although the AUCs of 200 mg BID (MTD) and 400 QD were comparable, 400 mg QD was not tolerated, which may be ascribed to the enhanced C_max_. As the T_1/2_ of LY2880070 was as short as 5.35 ± 2.3 hours, the median steady state C_min_ of 200 mg BID schedule was enhanced and remained above IC_50_ for 24 hours. However, the best response of LY2880070 monotherapy was stable disease in 16% patients. LY2880070 was also explored by combination with low dose gemcitabine in advanced/metastatic HGSOC patients^243^ (Table 6). The RP2D of LY2880070 in this scenario was 50 mg BID days 1 - 5 weekly, which is more intensive than the MTD as monotherapy. As of data reported, 59.3% patients achieved disease control but the ORR was only 7.3%. Now the clinical study of LY2880070 combined with low dose gemcitabine in genetically selected HGSOC subpopulation is conducted by Esperas Pharma. Thus for both prexasertib and LY288070, new biomarkers for patient selection is of extremely importance in future clinical trials.

Recently, a new oral and CHK1 selective inhibitor XS-02 was disclosed.^244^ In a cell-based CHK1 enzymatic activity analysis, XS-02 showed comparable potency with prexasertib but more potent than LY2880070 and SRA737. In vivo, XS-02 illustrated meaningful antitumor effect in several xenograft models either as single agent or by combination with a PARP inhibitor. XS-02 demonstrated favorable bioavailability and safety profile across species. All in all XS-02 is a new oral CHK1 selective inhibitor with improved potency than LY2880070 and SRA737. The IND filing of XS-02 is expected in second half of 2023.

### PKMYT1 inhibitors

PKMYT1 also belongs to WEE1 kinase family that mediates the inhibitory phosphorylation of CDK1^245^ (Fig. 4). Albeit PKMYT1 and WEE1 are seemingly redundant in negative regulation of CDK1, there are several major discrepancies:^246^ (i) WEE1 phosphorylates both CDK1 and CDK2 at Tyr15 but PKMYT1 only phosphorylates CDK1 at Thr14; (ii) WEE1 is mainly nuclear-localized, while PKMYT1 is cytoplasmic via a membrane-tether to endoplasmic reticulum and Golgi complex; (iii) PKMYT1 could sequester CDK1 to prevent its entry into nucleus. Importantly, it seems that PKMYT1 was dispensable for normal cell cycle progression, whereas WEE1 was somehow broad essential, given that *WEE1* knockout mice died of defective development.^247^

Recently, a genome-wide clustered regularly interspaced short palindromic repeat (CRISPR) knockout screen revealed that *PKMYT1* was synthetic lethal with *CCNE1* amplification in cancer cells.^248^ *CCNE1* amplification is prevalent in uterine, ovarian, stomach and other cancer types, which represent an unmet clinical need. Mechanistically, *CCNE1* amplification activated the transcription program MMB–FOXM1, which upregulated PKMYT1 substrate, cyclin B – CDK1 complexes. *CCNE1* amplification engendered replication stress and extended S phase. In light of these interesting findings, RP6306, a clinical-stage selective PKMYT1 inhibitor was developed^249^ (Fig. 6d). RP6306 demonstrated biased cytotoxicity to *CCNE1* amplification cancer cells, whereas adavosertib was both cytotoxic irrespective of *CCNE1* background. RP6306 treatment resulted in activated CDK1, premature mitosis entry and DNA damage, which is reminiscent of WEE1 inhibition by adavosertib. In xenograft animal models harboring *CCNE1* amplification or *FBXW7* (encode the E3 ubiquitin ligase which degrades CCNE1) loss, RP6306 dramatically inhibited tumor growth either as monotherapy or in combination with gemcitabine. Now the ongoing clinical trials of RP6306 recruit patients with *CCNE1* amplification or *FBXW7* loss. We wonder whether the different characteristics of PKMYT1 and WEE1 could bring about a wider therapeutic index for RP6306 than adavosertib in clinic.

### PLK1 inhibitors

PLK1 is the best studied member of human polo-like serine/threonine kinase family. Like other PLKs, PLK1 is comprised of a *C*-terminal polo-box domain (PBD) and an *N*-terminal kinase domain.^250,251^ PBD domains aid in the localization and substrate recognition of PLK1 within cell. To achieve full activation, PLK1 needs to be phosphorylated by upstream kinase Aurora-A and its cofactor Bora at threonine 210 within T-loop (Fig. 4). The best known physiological function of PLK1 is its role in G2/M phase, including timing of mitotic entry and exit, centrosome regulation, coordination of spindle assembly, correct chromosomal segregation and cytokinesis.^252^ PLK1 expression is exquisitely regulated throughout cell cycle: upregulated in G2/M phase while keep at low level in interphase.^253^ PLK1 function in the course of DNA replication,^254^ DDR^255^ and DNA damage associated cell cycle checkpoint^256^ has only been unveiled in last a few years. During replication and especially replicative stress, PLK1 phosphorylates a subset of substrates including origin recognition complex 2 (ORC2), minichromosome maintenance complex 2-7 (MCM2-7) and other components to regulate licensing and firing.^257^ At the end of replication, cyclin-B1/CDK1 complex facilitates the Aurora-A/Bora complex formation, which in turn activates PLK1. PLK1 then mediates inhibitory phosphorylation on WEE1 and PKMYT1 to promote their degradation and further activation of CDK1^258^ (Fig. 4). These intertwined feedback loops guarantee the smooth transition from DNA replication to mitosis. In the presence of DNA damages, ATM and ATR mediate phosphorylation and degradation of Bora, which will inhibit PLK1 activity.^259^ Moreover, PLK1 is also implicated in HR process,^260,261^ epithelial to mesenchymal transition (EMT),^262^ autophagy,^263^ apoptosis^264^ and even inflammatory response.^265^ These versatile functions are closely related to cancer initiation and progress, which make PLK1 an attractive target for cancer treatment.^266^ Two strategies have arisen for the development of PLK1 inhibitors, either targeting PBD domain or kinase domain. Right now, only ATP-competitive inhibitors are under active clinical development.

Volasertib (also known as BI6727, developed by Boehringer Ingelheim) is the most advanced ATP-competitive PLK1 inhibitor in clinic.^267^ Of note, volasertib potently inhibited PLK1 as well as PLK2 and PLK3, even though to a lesser extent. As PLK2 and PLK3 may function as tumor suppressors,^268^ this may conflict the antitumor effects of volasertib induced by PLK1 inhibition. In vitro, volasertib showed broad antiproliferation effect in cancer cell lines by inducing G2/M arrest and apoptosis. With favorable intravenous pharmacokinetic profile and high volume of distribution, volasertib demonstrated meaningful in vivo efficacy either as monotherapy or by combination with chemotherapy or radiation.^269^ The RP2D of volasertib monotherapy in patients was determined at 300 mg per administration every 3 weeks.^270^ As expected, the most frequent AEs were hematological toxicities including anemia, neutropenia, and thrombocytopenia. However, most reported clinical efficacies of volasertib monotherapy or in combination with other agents in solid tumors were less optimal.^271^ Though early results of volasertib in combination with low-dose cytarabine (LDAC) in acute myeloid leukemia (AML) patients seems intriguing,^272^ a following large phase 3 trial failed to reproduce the positive results.

Different from volasertib, onvansertib (also known as NMS1286937, developed by Cardiff Oncology) is an oral and potent ATP-competitive PLK1 inhibitor with high selectivity over PLK2 or PLK3.^273,274^ Onvansertib also demonstrated broad antiproliferation effects and produces remarkable in vivo efficacy either as single agent or in combination with chemotherapy. In patients, the MTD and RP2D of onvansertib was determined to be 24 mg/m^2^/day in 5 consecutive dosing followed by a 16-day holiday.^275^ The monotherapy DLTs were mainly thrombocytopenia and neutropenia, which are consistent with volasertib. Impressively, onvansertib adopted different strategies in the following clinical trials. Consistent with finding from a genome-wide RNA interference (RNAi) screen which identified that PLK1 inhibition is synthetic lethal with *KRAS* mutation,^276^ onvansertib showed a biased cytotoxicity to cells carrying *KRAS* mutation compared to wildtype isogenic.^277^ As a result, onvansertib is explored in a combination trial with folinic acid, 5-fluorouracil, and irinotecan (FOLFIRI) and bevacizumab (a VEGFR antibody) for treatment of 2^nd^ line *KRAS* mutant colorectal cancer patients. According to a recent report, the ORR, DCR and mPFS were determined to be 35.4%, 91.7% and 9.3 months respectively, all remarkably better than historical data.^278^ Of note, the response rate in *KRAS* responders (≥ 90% decrease in KRAS mutant allele frequency in circulating tumor DNA (ctDNA) after 1 cycle of treatment) was considerably higher than that of *KRAS* nonresponders. As *KRAS* mutation hints replication stress, we anticipate that the role of PLK1 in replication may be the underlying mechanism of synthetic lethal relationship. Now onvansertib is also explored by combination with other agents in clinic (Table 2). We are looking forward to more mechanistic studies of PLK1 in disease condition to help patient selection in future.

### Aurora-A inhibitors

As the upstream regulator of PLK1, Aurora-A is also an attractive antitumor target.^279^ Aurora-A, as well as Aurora-B and Aurora C, all belong to Aurora serine/threonine kinase family. These 3 paralogues share a conserved *C*-terminal kinase domain but the *N*-terminal domains are varied.^280^ Upon activation, Aurora kinases will auto-phosphorylate themselves on catalytic T-loop residues. Aurora kinases are all implicated in cell division: Aurora-A is responsible for centrosome maturation and segregation, and spindle assembly in mitosis;^281^ Aurora-B coordinates microtubule attachments to centrosome and phosphorylates histone H3 (pHH3) in mitosis;^282^ whereas Aurora-C is mainly expressed in testis and involves in meiosis and embryonic development.^283^ The different physiological functions of Aurora kinases suggest the necessity for developing selective Aurora-A inhibitors. As aforementioned, Aurora-A can also regulate mitotic entry (Fig. 4). In addition to activating PLK1, Aurora-A also mediates phosphorylation of BRCA1 at serine 308 to promote G2/M transition.^284^ Moreover, the inhibitory phosphorylation of p73 at serine 235 by Aurora-A leads to abrogation of DNA damage induced apoptotic response and mitotic spindle assembly checkpoint (SAC).^285^ Like PLK1, Aurora-A expression peaks in G2/M phase but decays in interphase in normal cells. However, Aurora-A overexpression is observed in numerous cancer types irrespective of cell cycle phases.^286^ In cancer cells, Aurora-A suppresses apoptosis and autophagy, activates the Wnt/β-catenin signaling pathway and promotes EMT.^287^ Of note, Aurora-A inhibition is synthetic lethal with tumor suppressor gene deficiencies such as *RB1*, *SNF5*, *SMARCA4* or *ARIDA1A.*^286^ Interestingly, Aurora-A is also associated with resistance to EGFR^288^ or PI3K-mTOR-Akt^289^ pathway inhibitors, and the addition of Aurora-A inhibitors can circumvent the resistance in preclinical studies. In KRAS^G12C^ mutant tumor cells, Aurora-A facilitates the interaction between KRAS and C-RAF and is associated with adaptive reactivation of KRAS after KRAS^G12C^ inhibitor treatment.^290^ Combination of an Aurora-A inhibitor and a KRAS^G12C^ inhibitor shows synergistic effect in vitro and in vivo. All these evidence makes Aurora-A an attractive antitumor target.

Alisertib (also known as MLN8237, developed by Millennium) is the most advanced oral and ATP-competitive Aurora-A inhibitor in clinical-stage.^291,292^ Alisertib showed > 200 fold selectivity over Aurora-B either in both enzymatic and cell based assays. Treatment of alisertib resulted in delayed mitotic entry, accumulation of tetraploid (4N) cells and M phase cells with abnormal mitotic spindles and misaligned chromosomes, which were consistent with Aurora-A physiological functions. Alisertib moderately inhibited or even suppressed in vivo tumor growth in models covering solid tumors and lymphoma. Importantly, even at in vivo MTD dosage, Aurora-B was not inhibited at all as illustrated by no changes in pHH3 in vivo.^291^ The RP2D of alisertib as single agent in clinic was determined to be 50 mg BID 7 days on/14 days off.^293^ The main DLTs of alisertib were fatigue, nausea, neutropenia, and stomatitis. Stomatitis may be correlated with benzodiazepine-like structure of alisertib, but not Aurora-A inhibition itself.^294^ Although with some promising results in several phase1/2 studies, alisertib alone failed to show superiority with respect to efficacy in a large phase 3 clinical trial when compared to chemotherapy for the treatment of peripheral T-cell lymphoma (PTCL) as a single agent.^295^ Of note, the rates of severe adverse events appeared comparable in both arms. Alisertib was also explored by combination with chemotherapy or other targeting therapy in clinic. However, most of the combinations discontinued or failed due to limited efficacy or intolerability.^294^ Of note, preliminary results of osimertinib plus alisertib in osimertinib-resistant NSCLC patients was disclosed.^296^ The benefit in this arm was inferior to another combination of osimertinib and sapanisertib (an mTOR inhibitor), although the TEAEs were comparable for both arms. Overall, it seems difficult to balance risk and benefit for alisertib in patients. Concerning that another highly selective Aurora-A inhibitor LY3295668 has been discontinued,^297^ we suspect that future development of Aurora-A inhibitors requires more thorough understanding of the role of Aurora-A in tumors.

### p53 Y220C reactivators

As the best known tumor suppressor, p53 (encoded by *TP53*) regulates transcription of a spectrum of genes involved in genome integrity maintenance, cell cycle checkpoint, apoptosis and other physiological processes.^298,299^ Upon DNA damage, p53 would be phosphorylated and activated by ATR, ATM, CHK1 or CHK2, leading to cell cycle arrest (Fig. 4), DDR gene expression or cell death. *TP53* mutation are frequently found in almost 50% of tumor patients.^300^ These mutations either disrupt the binding to DNA or destabilize p53, and eventually attenuation of p53 function in transcription regulation.^301,302^ Generally loss of function of tumor suppressors is difficult to target directly, by alternative synthetic lethal strategies are readily employed in these conditions, which is exemplified by ATR or WEE1 inhibitors in *TP53* deficient tumors. However in the cases of *TP53* mutation, several small molecule reactivators which can restore p53 function have proceeded to clinical trials.^303,304^ In particular, hot spot mutation Y220C is amenable for selective reactivator development.

p53^Y220C^ accounts for around 1.8% of all p53 mutations, and is broadly detected across various solid tumor types.^300^ Unlike other hot spot mutations, Y220C locates distant away from DNA binding interface of p53. Y220C inactivates p53 by destabilization of p53 DNA binding domain by around 4 kcal/mol.^305^ Of the most importance, Y220C left a cavity on p53 surface which can be bound by small molecules.^306^ A set of binders have been developed,^307–309^ which promoted p53^Y220C^ stability, restored conformation and transcription regulation, and selectively led to *TP53*^*Y220C*^ mutant tumor cell apoptosis. In this sense, p53^Y220C^ may be a promising tumor agnostic target.

PC14586 is the first clinical-stage selective p53^Y220C^ reactivator. In preclinical evaluations, PC14586 restored p53^Y220C^ to wildtype conformation, induced p53-regulated gene expression such as p21 and MDM2, and regressed *TP53*^*Y220C*^ mutant tumor in vivo.^310^ Remarkably, in an engineered mouse model carrying *TP53*^*Y220C*^ mutation, PC14586 combined an anti-PD1 antibody led to 6 out of 10 complete response and dramatically extended median survival time.^311^ Recently, preliminary response was reported in patients harboring *TP53*^*Y220C*^ mutation during PC14586 dose escalation.^312^ PC14586 reached maximal tolerated dose at 1500 mg BID with acceptable safety profile. Now the clinical study is still ongoing to determine RP2D for PC14586 (Table 2).

## Other DDR targets on the rise

### Polθ

Polθ, a 290 kDa protein which contains an *N*-terminal helicase-like domain (HelD) and a *C*-terminal polymerase domain (PolD), serves the predominate role in TMEJ^91,100^ (Fig. 3). Both TMEJ and HR require DSB end resection, whereas unlike HR was only active in the presence of homologous chromatins as template, Polθ can be facilitated by even < 5 bp microhomology in ssDNA overhangs.^86^ Polθ HelD is an ssDNA-activated ATPase and serves to remove ssDNA-bound RPA, while PolD was responsible for DNA synthesis from microhomology sites. Albeit TMEJ is intrinsically error-prone compared to HR, TMEJ rendered cell survive by avoiding more catastrophic genome aberrations.^313^ Recently, *POLQ* down regulation was shown to be synthetic lethal with a group of other DDR gene deficiencies, including those from HR and NHEJ.^314^ Hence TMEJ is reckoned as the salvage pathway to deficient HR or NHEJ, and Polθ inhibitors was exploited in these conditions, especially HRD.^315^

Novobiocin^316^ and ART558,^317^ which inhibit HelD and PolD, respectively, represent two strategies to disrupt Polθ function. Both novobiocin and ART558 phenocopied *POLQ* selective dependency in HRD cancer cells. Interestingly, loss of functional 53BP1/Shieldin complex in HRD cells conferred resistance to PARP inhibition, but hypersensitive to Polθ inhibitors.^317^ Concerning 53BP1 and Shieldin complex channeled NHEJ by preventing DSB end resection, these findings suggested that end resection is indispensable for DSBR choice towards TMEJ. Of note, novobiocin circumvent PARPi resistance in HRD PDX models by monotherapy or in combination with PARP inhibitors. RP6685 was another PolD inhibitor reported by Repare, which selectively killed *BRCA2* knockout cancer cell line compared to wildtype isogenic.^318^ RP6685 enhanced micronuclei and DNA damage marker γH2AX in *BRCA2* knockout tumor models. These studies validate the potential of Polθ inhibition in clinic, however targeting which domain will be better is not conclusive so far.

Recently, the first in class Polθ PolD inhibitor ART4215 initiated a phase 2 clinical trial in combination with talazoparib for the treatment of *BRCA* deficient breast cancer (Table 2). For Polθ HelD domain, Ideaya disclosed an inhibitor, which displayed synergistic effect with niraparib in *BRCA* deficient model. The first-in-human study of Ideaya’s compound is expected in 2023.

### RAD51

RAD51 recombinase is an ATPase and functions as a critical effector in HR.^319^ After end resection at the DSB sites, the exposed ssDNAs are coated and protected by RPA. Subsequently BRCA2 in complex with RAD51 and other proteins displaces RPA, and RAD51 forms homopolymeric filaments with ssDNA (Fig. 3). Then RAD51 nucleoprotein filament conducts homology search and strand invasion to a sister chromatin, and use it as the template for DNA synthesis. Additionally in cells struggling with replication stress, RAD51 promotes replication fork reversal, inhibits fork degradation and orchestrates break induced replication (BIR).^320^ Of note, RAD51 strand exchange activity is required for HR and BIR but dispensable for replication fork reversal. Mutations in *RAD51* as one type of HRD are related to cancer susceptibility and FA-like syndromes.^321^ In brief, RAD51 was essential for genome integrity. Different modes of inhibition have been reported for RAD51, including ssDNA binding disruption, oligomerization interference, and inhibition to D-loop formation.^322–324^ All these RAD51 inhibitors showed antiproliferation effect in a range of cell lines and potentiate other antitumor drugs such as cisplatin and topoisomerase inhibitors.

To our knowledge CYT0851 remains the only clinical-stage compound claimed as RAD51 inhibitor (Table 2), albeit the precise mode of inhibition was still undisclosed.^325,326^ Interestingly, CYT0851 was recently verified as an inhibitor of monocarboxylate transporter which medicates monocarboxylated biomolecules transportation. CYT0851 was optimized from hits identified through a phenotypic screen, which selectively inhibited high activation inducted cytidine deaminase (AID) expression cancer cell growth but spared normal cells with low AID expression. AID stochastically deaminates cytidines throughout genome, leading to point mutation, SSBs and DSBs.^327^ Ectopic AID is broadly expressed in multiple solid tumor types and nonhodgkin lymphoma (nHL), which confers dependency on HR and RAD51. In AID positive cells, CYT0851 reduced RAD51 foci formation and induced γH2AX expression, showed >30 fold selectivity over *AID* knockout cells. In vivo CYT0851 suppressed AID positive tumor growth and potentiate a PARP inhibitor. Recently, the clinical data of CYT0851 dose escalation study was reported.^328^ As a single agent, CYT0851 displayed favorable PK and safety profile, and preliminary response was observed in heavily pre-treated patients, especially nHL. Now the RP2D has been determined, meanwhile dose expansion and combination study is still ongoing (Table 2).

### USP1

USP1 in complex with UAF1 is one type of deubiquitinases that regulates FA and TLS through deubiquitination of several platform proteins in these DDR processes^329^ (Fig. 2d). For instance during FA for the response to interstrand crosslinks induced by platinum drugs, USP1 deubiquitinates monoubiquitinated FANCD2 (ub-FANCD2) and FANCI (ub-FANCI), either of which acts as a scaffold to recruit other repair proteins. While in TLS and DNA replication, USP1 mediates deubiquitination of monoubiquitinated proliferating nuclear antigen (PCNA) which would disrupt unscheduled recruitment of error-prone TLS polymerases such as Polκ and REV1.^330^ Both Polκ and REV1 would introduce single nucleotide mutations and cause genome instability, in this sense USP1 serves a protective role for genome integrity.

Recently, a genome-wide CRISPR knockout screen identified *USP1* to be selective essential in breast & ovarian cancer cell lines harboring HRD, especially *BRAC1/2* deficiency.^331^ It is prospected that *BRCA1* deficient cells was characterized by fork instability and cannot tolerate more instabilities by the absence of active USP1.^330^ By mechanism persistent monoubiquitinated PCNA was responsible for synthetic lethal relationship between USP1 and BRCA1. Thus USP1 inhibitor was anticipated to be effective either as monotherapy in *BRCA* deficient cells or by combination with other DNA damaging agents including platinum drugs. Interestingly, USP1 with defective autocleavage activity cannot recycle itself from DNA, a phenomenon called USP1 trapping.^332^ But to our knowledge there has been no USP1 inhibitor claimed USP1 trapping capability so far.

The first in class clinical-stage USP1 inhibitor, KSQ4279, is highly potent (Ki: 1.2 nM) and selective over other deubiquitinases. Interestingly, kinetic analysis indicated that KSQ4279 is allosteric and substrate uncompetitive. KSQ4279 demonstrated hyperactive in cancer cells with HRD or *BRCA* deficiencies in vitro and in vivo.^331^ Of note, KSQ4279 led to cell cycle arrest, accumulated DNA damage and replication fork degradation in *BRCA1* deficient cells, which is consistent with USP1 biological functions. A CRISPR screen revealed that *PCNA* loss was associated with KSQ4279 resistance but loss of BER genes including *PARP1* conferred hypersensitivity to KSQ4279. Particularly, KSQ4279 potentiate olaparib in PARPi insensitive or partially resistant PDX models. Now KSQ4279 was evaluated in a phase 1 clinical trial (Table 2).

### PARG

In contrary to PARP, PARG is responsible for the catabolism of PAR^333^ and consequently the release of DNA repair complex from genome. Lines of evidence suggest protective roles of PARG in SSBR (Fig. 2a), DSBR and especially replication. As dynamic and stringent regulation of PARylation is indispensable for optimal DDR, PARG is also validated as a potential DDR target for cancer treatment.^334^ PARG inhibition was shown to be hypersensitive in cells with replication vulnerability, leading to failure to restart stalled replication fork and persistent replication stress.^335^ Although a set of PARG inhibitors have been reported, none of them reaches clinic to our knowledge. Recently, a clinical candidate PARG inhibitor IDE161, was disclosed by Ideaya. Accordingly, IDE161 behaved a different profile than PARP inhibitors in a panel of cancer cell lines irrespective of HRD, suggesting different dependency of PARG and PARP in cancers. In vivo IDE161 can impressively regress tumor growth even in PARPi-resistant PDX models. IDE161 is expected to enter clinic soon.

### WRN

WRN helicase is recently identified as an intriguing synthetic lethal target in dMMR or microsatellite instability high (MSI-H) cancers.^336–338^ In dMMR/MSI-H cancer cells featured by (TA)_n_-dinucleotide repeat expansions, WRN could unwind non-B DNA cruciform-like structures formed by (TA)_n_ repeats during replication. Otherwise without the presence of functional WRN, the replication fork will be stalled at cruciform-like structures and resulted in DSBs and apoptosis. Given that DNA cruciform-like structures were only detected in MSI-H tumors, WRN is presumed collateral essential in MSI-H rather than MSS (microsatellite stable) tumors. Meanwhile in *BRCA2* deficient cells, WRN compensated BRCA2 function in safeguarding genome stability through rescuing the stalled replication forks and suppressing MRE11-mediated fork degradation.^339^ These cumulative evidence suggest the potential of WRN as a synthetic lethal target and prompt a certain of medicinal efforts for the development of WRN inhibitors.^340^ However to our knowledge, none of WRN inhibitors has proceeded into clinic hitherto.

## Conclusion and future perspective

With decades of antitumor innovations, small molecule drugs such as chemotherapy and targeted therapy have dramatically changed cancer treatment paradigm.^341–343^ Chemotherapy drugs unbiasedly attack essential substances, leaving a narrow or even inverted therapeutic index (TI) in patients. The TIs of targeted therapy are generally high, due to selective targeting oncogenic gene aberrations within cancer cells (*EGFR*, *KRAS*, etc), or genes essential in restricted lineages (*BTK*, *BCL2*, etc). Another high-TI example is synthetic lethal, which is prevalent within DDR genes exemplified by selective essential of *PARP1* in *BRCA* deficiency or HRD. However, ATR, CHK1 and WEE1 appear to be broad essential for cancer cells across various histological origins.^344^ For these targets, development strategies cannot simply copy conventional targeted therapy, somehow even akin to chemotherapy. Although the essentiality of DDR genes are different, there are several commonalities can be compiled to enhance the probability of success for DDR targeting therapy.

(i) Enhance selectivity to mitigate off-target toxicities. Dissecting that PARP1 and PARP2 behave differently in the contributions to efficacy and hematological toxicity, PARP1 selective inhibitors with reduced toxicity also achieved higher TEC in clinic.^146^ Likewise, azenosertib can be dosed continuously with more manageable safety profile compared to adavosetib partially in that azenosertib was more selective in the kinase selectivity profile.^197,200^ Hence selectivity enhancement can not only reduce off-target toxicity but deepen or prolong on-target inhibition.

(ii) Identify predictive biomarker. The success of PARP inhibitors showcased the power of predictive biomarker. New DDR targets *USP1*, *PKMYT1*, and *WRN* were identified in given gene aberrations. In preclinical evaluations, cell line panel, PDXs, and organoids enable the characterization of responders and nonresponders for a DDR inhibitor.^345^ For the existed clinical-stage CHK1/2 inhibitor prexasertib, a companion diagnostic test has been employed to select patients in a newly initiated clinical trial (NCT05548296) (Table 2). Concerning on-target toxicity is almost unavoidable, a new mechanism or drug with clear predictive biomarker may help stratify and enrich clinical trial population, so as to widen the therapeutic index.

(iii) Combination with more cancer hallmarks and modalities. Combinations seems to be a permanent topic for DDR targeting therapy. Typically DDR inhibitors were combined with chemotherapy or other DDR inhibitors, which would magnify genome instabilities, lead to enhanced efficacy as well as toxicity. Recently, olaparib plus bevacizumab significantly prolonged mPFS as first line maintenance therapy in ovarian cancer patients with HRD (PAOLA-1 trial).^346^ The addition of olaparib did not increase the known toxicity of bevacuzumab. In a similar vein, olaparib in combination with abiraterone (a CYP17 inhibitor) boosted benefit for first line metastatic castration-resistant prostate cancer, also with no additional toxicities compared to either drug alone (PROpel trial).^347^ These evidence hints that DDR targeting therapy can exploit combinations with drugs targeting different cancer hallmarks with nonoverlapped toxicities. Antibody drug conjugate (ADC) can be regarded as tumor-oriented delivery of chemotherapy. Trastuzumab deruxtecan (T-DXd), an ADC composed of anti-HER2 antibody and a cytotoxic topoisomerase I inhibitor, showed synergistic effect both in vitro and in vivo by combination with ceralasertib, adavosertib, AZD1390 or AZD5305.^348,349^ Of note, no synergistic interaction was observed in the in vitro human bone marrow assay treated with T-DXd combined with ceralasertib or adavosertib. The localized cytotoxic effect of ADC may warrant further investigation in combination with DDR targeting therapy (Table 2).

(iv) New target identification and evaluation. By using phenotypic genome-wide CRISPR knockout screen, new synthetic lethal gene *PKMYT1* and *USP1* were identified in the condition of *CCNE1* amplification and HRD, respectively. It is notable that in recent years CRISPR knockout screen has been broadly applied in the search of synthetic lethal pair, sensitive or resistant biomarkers and new target opportunities.^350^ The gene essentiality analysis is necessary for the prediction of efficacy and toxicity. Nowadays Cancer Cell Line Encyclopedia (CCLE),^351^ The Cancer Genome Atlas (TCGA)^352^ and other databases have empowered the essentiality evaluation in normal tissues, restricted lineages and cancer cells. In particular, the DDR gene associated hereditary disease can also provide a path to delineate physiological function and clinical scenarios for DDR targets.^353^

As cancer remains as one of the top health threats to humanity, new MOAs (mechanism of actions) and drugs are still of great requirements to improve cancer prognosis. Inspired by the precedent success and thriving advancements, we believe the new wave of innovations targeting DDR network will open up new opportunity to expand the toolkit for antitumor treatment.
